# Novel TPLO Alignment Jig/Saw Guide Reproduces Freehand and Ideal Osteotomy Positions

**DOI:** 10.1371/journal.pone.0161110

**Published:** 2016-08-24

**Authors:** Abigail D. Mariano, Michael P. Kowaleski, Randy J. Boudrieau

**Affiliations:** Department of Clinical Sciences, Cummings School of Veterinary Medicine, Tufts University, North Grafton, Massachusetts, United States of America; University of Bari, ITALY

## Abstract

**Objectives:**

To evaluate the ability of an alignment jig/saw guide to reproduce appropriate osteotomy positions in the tibial plateau leveling osteotomy (TPLO) in the dog.

**Methods:**

Lateral radiographs of 65 clinical TPLO procedures using an alignment jig and freehand osteotomy performed by experienced TPLO surgeons using a 24 mm radial saw blade between Dec 2005–Dec 2007 and Nov 2013–Nov 2015 were reviewed. The freehand osteotomy position was compared to potential osteotomy positions using the alignment jig/saw guide. The proximal and distal jig pin holes on postoperative radiographs were used to align the jig to the bone; saw guide position was selected to most closely match the osteotomy performed. The guide-to-osteotomy fit was categorized by the distance between the actual osteotomy and proposed saw guide osteotomy at its greatest offset (≤1 mm = excellent; ≤2 mm = good; ≤3 mm = satisfactory; >3 mm = poor).

**Results:**

Sixty-four of 65 TPLO osteotomies could be matched satisfactorily by the saw guide. Proximal jig pin placement 3–4 mm from the joint surface and pin location in a craniocaudal plane on the proximal tibia were significantly associated with the guide-to-osteotomy fit (*P* = 0.021 and *P* = 0.047, respectively).

**Clinical Significance:**

The alignment jig/saw guide can be used to reproduce appropriate freehand osteotomy position for TPLO. Furthermore, an ideal osteotomy position centered on the tibial intercondylar tubercles also is possible. Accurate placement of the proximal jig pin is a crucial step for correct positioning of the saw guide in either instance.

## Introduction

One of the more common surgical procedures in the dog for treatment of the cranial cruciate ligament deficient stifle joint is the tibial plateau leveling osteotomy (TPLO). The TPLO employs a radial osteotomy of the proximal tibia and subsequent rotation of the tibial plateau to accomplish a 5° tibial plateau angle (TPA) in order to neutralize cranial tibial thrust [1]. The original alignment jig (Slocum Enterprises, Eugene, OR; USA), which is secured to the proximal and distal tibia, was developed to maintain a fixed plane orientation of the tibial segments during rotation. The choice of osteotomy location, however, is independent of the jig position and performed freehand by the surgeon.

Proper angulation of the osteotomy is an important aspect of the procedure so as to avoid a number of unwanted alterations of tibial alignment and torsion after rotation of the proximal tibia [2]. Osteotomy positioning is critical in order to minimize stress on the tibial tuberosity, which may result in a fracture [3]. Attaining the target TPA post-rotation is also dependent on the osteotomy position on the tibia as distal positioning can result in a higher than expected tibial plateau angle and persistent cranial tibial thrust [4,5]. Subjective guidelines were originally established to avoid these technical errors: orientation of the osteotomy parallel to the joint surface and perpendicular to the sagittal plane of the tibia, and placement sufficiently caudal to preserve tibial tuberosity bone support [1]. Furthermore, more recently, objectively formulated recommendations have been proposed, including centering the osteotomy over the tibial intercondylar tubercles on the long axis of the tibia, and leaving ≥10 mm of bone at the level of the tibial tuberosity in the medium to large breed dogs [3,4,6]. Lastly, the osteotomy necessitates a footprint only sufficiently large to accommodate the head of the plate such that the screws placed to secure it are not directed intra-articularly and/or in the osteotomy [3,6,7]. Despite knowledge of these guidelines, the technical aspect in accurately executing these aspects of the technique may be difficult, particularly for novice surgeons. Accurate positioning and angulation of the osteotomy can be difficult to perform; in addition, initiation of the osteotomy is especially challenging given the irregular shape of the proximal tibia and tendency for the saw to “walk” on the sloped medial cortex.

**Fig 1:**
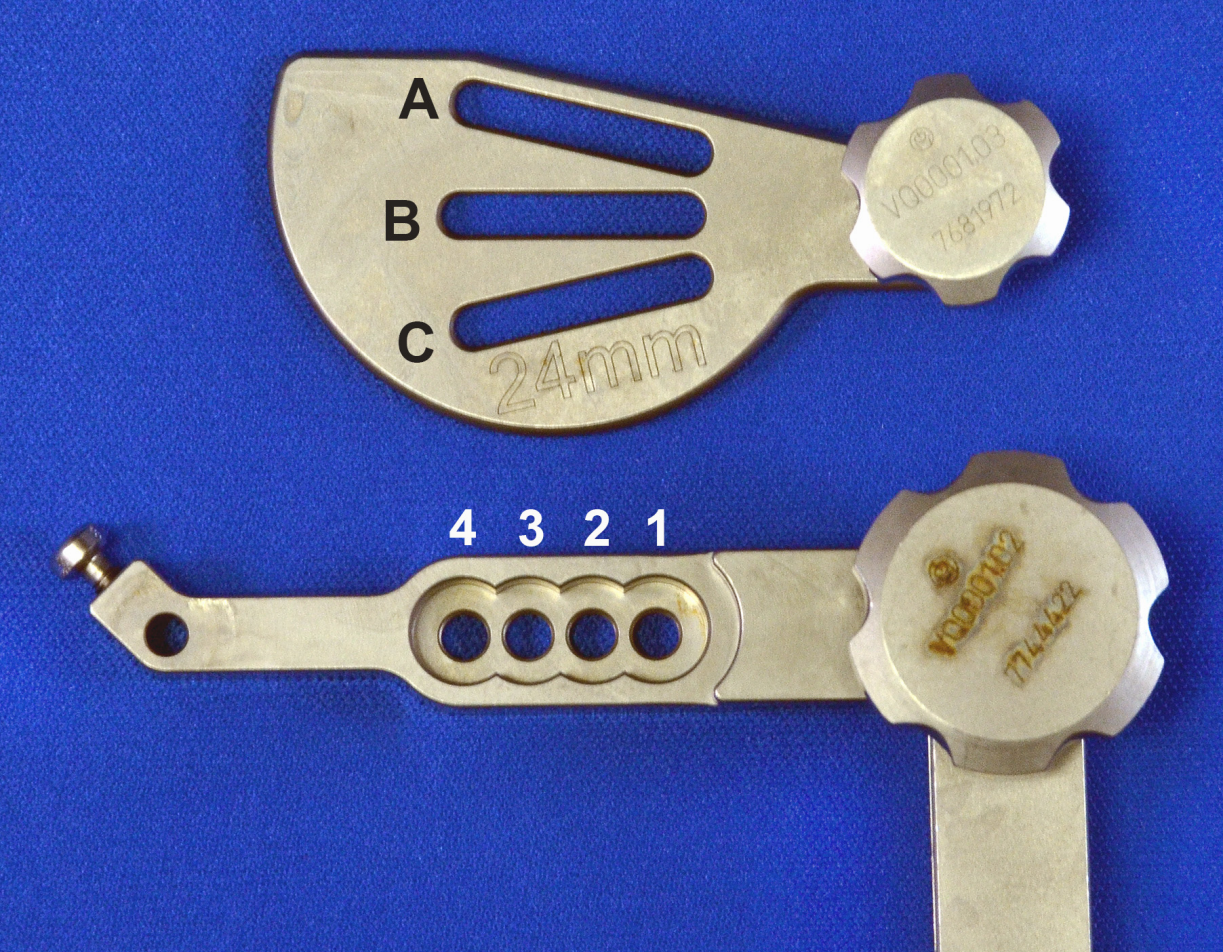
The DePuy Synthes TPLO saw guide has a combination of 12 possible positions for attachment to the jig; three angular positioning slots on the guide for the jig pin (A, B, C) and four bolt hole choices for securing the guide to the jig (1, 2, 3, 4).

In 2009, a TPLO alignment jig with an adjustable 24, 27, and 30 mm radial saw guide attachment (DePuy Synthes^®^ Vet, West Chester, PA; USA) for use during the TPLO procedure became commercially available (Fig 1). In an *in vitro* experimental model, this alignment jig/saw guide was demonstrated to produce cuts more orthogonal to the tibial long axis, with less damage to the medial cortex from “skidding” compared to osteotomies made with the alignment jig alone [8]. Additionally, the osteotomies made with the aid of the saw guide were found to be significantly more accurate in matching the intended osteotomy positions [8]. In a subsequent retrospective clinical study, osteotomies made using the alignment jig/saw guide had more accurate centering of the osteotomy and less deviation from the target postoperative TPA compared to osteotomies performed with the alignment jig only [9]. Using the saw guide for osteotomy positioning has not been demonstrated, however, to accurately replicate the position obtained by freehand osteotomy by experienced surgeons in clinical cases, nor its ability to locate the osteotomy in the ideal osteotomy location (IOL) as was initially described by Kowaleski [4]. Furthermore, since the inception of the TPLO procedure, the proximal jig pin location recommendations have changed in relation to the medial collateral ligament (MCL),[1,7,9] which also will have an effect on the osteotomy position using this alignment jig/saw guide, since the jig and saw guide are attached to the jig pin (Slocum B. Tibial plateau leveling osteotomy course. Eugene, OR, Slocum Enterprises Inc. 1998).

The objective of this study was to review freehand clinical TPLO jig pin and osteotomy locations, as performed by experienced TPLO surgeons, and additionally the IOL, and evaluate the complementary position that could be obtained using the alignment jig/saw guide. Our hypothesis was that when referenced from the proximal jig pin, the alignment jig/saw guide could accurately reproduce the desired and appropriate osteotomy positions performed freehand by surgeons experienced with the TPLO technique, and secondly, would also result in the ability to match the IOL from this same reference jig pin. The ultimate objective was to demonstrate that the simulated use of the saw guide/jig pin combination could successfully replicate a range of appropriate osteotomy positions in clinical cases, thus establishing that this instrumentation could be used by inexperienced surgeons to successfully replicate the position produced by experienced surgeons.

## Materials and Methods

### Case Selection

The hospital’s electronic medical record database was used to identify dogs that underwent TPLO surgery between December 2005 and December 2007 (Group 1) and November 2013 and November 2015 (Group 2) at Cummings School of Veterinary Medicine at Tufts University. These two groups of cases were representative of the different proximal jig pin placement recommendations prevalent at each time frame (Slocum B. Tibial plateau leveling osteotomy course. Eugene, OR, Slocum Enterprises Inc. 1998) [1,7,9].

### Inclusion Criteria

For Groups 1 and 2, dogs of any breed, age, or sex with confirmation of cranial cruciate ligament insufficiency at surgery, operated on utilizing a 24 mm radius crescentic TPLO saw with an alignment jig (DePuy Synthes^®^ Vet; West Chester, PA; USA), and possessing standard mediolateral postoperative TPLO radiographs centered at the stifle and collimated to include the talocrural joint were candidates for the study. To be included, a board certified surgeon with experience of >300 previous TPLO surgeries must have performed the procedure with a freehand osteotomy. Dogs with bilateral TPLO procedures were reported as separate cases.

### Exclusion Criteria

Cases were excluded if radiographic positioning was not considered to be appropriate (>2 mm separation of the overlap of the femoral condyles) [10], or if jig pin holes could not be clearly identified on the mediolateral postoperative radiographs. During the timeframe of Group 2, the saw guide attachment became available; cases using the saw guide were excluded.

Appropriate osteotomies were defined as having a maximum displacement, in the x or y planes (placed along the anatomic axes of the tibia), of ≤7 mm (distance from the intended centroid of the osteotomy [ICO] of the IOL to the actual centroid of the osteotomy [ACO]; terminology consistent with previous study) [9], and a tibial tuberosity width ≥10 mm [3,6]. The x and y distances (including the resultant distance of eccentricity [DOE], resultant distance between the ICO and the ACO) and tibial tuberosity widths were determined as previously described [6,9].

### Radiographic Guide Measurements

**Fig 2:**
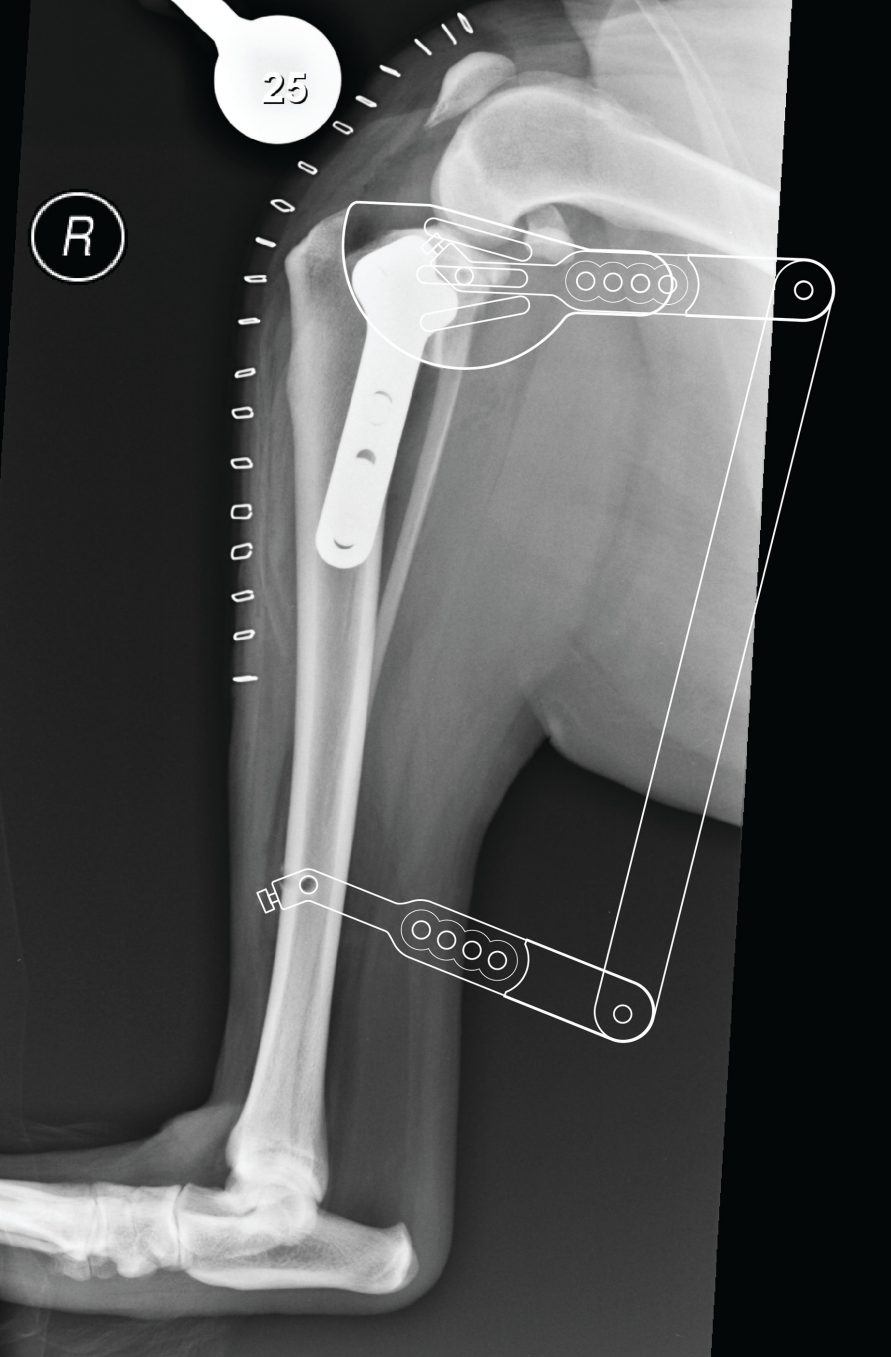
Digital representation of the method used to align the jig and guide onto a true-to-size printed postoperative lateral radiograph. The alignment jig/saw guide was aligned with the long axis of the tibia using the proximal and distal jig pin holes. The saw guide was then positioned to achieve the best fit over the osteotomy by angling the jig arms (see Fig 3). The widest offset along the radial osteotomy between the actual and saw guide osteotomy position was recorded (≤1 mm = excellent; ≤2 mm = good; ≤3 mm = satisfactory; >3 mm = poor).

**Fig 3:**
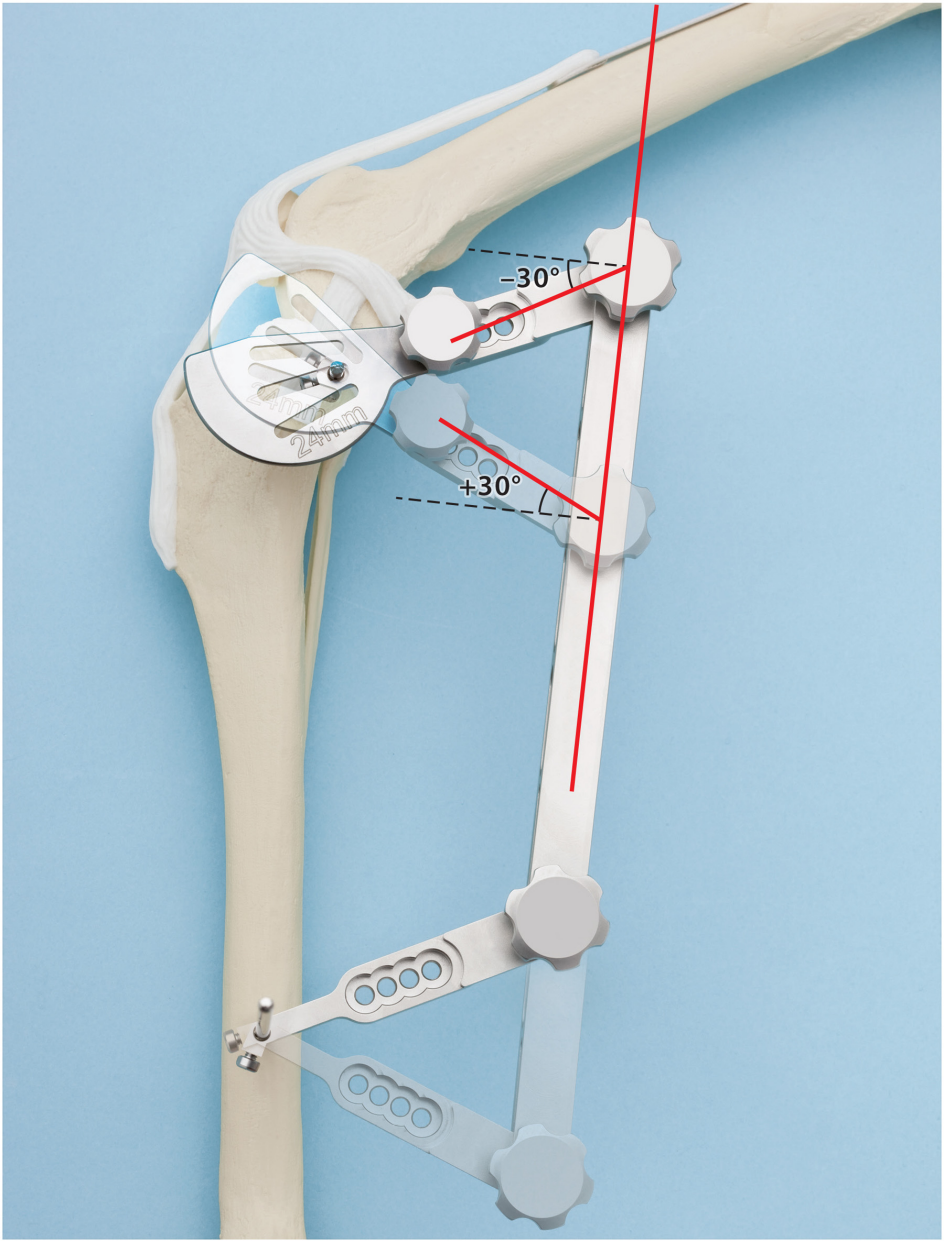
After the saw guide was placed in the closest position to the osteotomy, the jig arms were angulated to achieve the best guide-to-osteotomy fit. The baseline angle of the jig arm was 90°. Angles greater than 90° were recorded as positive and angles below 90° were recorded as negative. The magnitude of the angle was recorded as its total deviation from 90°. This image was reproduced from the DePuy Synthes Vet Technique Guide: “Standard Tibial Plateau leveling Osteotomy (TPLO) System” (J6544-C, 2013), with the courtesy of DePuy Synthes Vet, Inc. West Chester, PA; USA.

The lateral preoperative and immediate postoperative digital radiographs of the tibia centered on the stifle joint were calibrated and physical copies were printed, true to size, on Kodak DryView Laser Imaging Film (Carestream Health, Inc., Rochester, NY; USA) using a Kodak DryView 8900 printer (Carestream Health, Inc., Rochester, NY; USA). When calibration balls were not included in the films, the TPLO plate itself was used to calibrate the image for true to size image printing. Starting with the postoperative radiographs, each was mounted on a board and 3 mm pins were placed through the film corresponding to the proximal and distal jig pin holes on the radiograph. The saw guide and jig was attached and adjusted through all possible positions until the closest match to the osteotomy centered on the ACO was obtained, as illustrated with a radiograph and superimposed line drawing of the jig/saw guide (Fig 2). The saw guide position was selected from the 12 possible combinations available; four bolt hole choices for securing the saw guide to the jig (1, 2, 3, 4) and three angular positioning slots on the saw guide for the proximal jig pin (A, B, C) (Fig 1). In addition to the various guide attachment positions, the jig arm angles were adjusted by ±30° from a baseline of 90° (Fig 3). The angle range of ±30° was chosen as the upper/lower limit based on clinician experience for reasonable, conservative clinical applications (further excursion is possible depending on the jig pin location and selection of saw guide position). A tracing of the saw guide position and jig arm angle with the smallest offset from the actual osteotomy was made onto the radiograph. Offset was defined as the widest distance between this saw guide position and the best match to the freehand osteotomy. The offset was measured with a ruler, and the angle of the jig arm was measured with a standard goniometer. The guide-to-osteotomy fit was defined as excellent (≤1 mm offset), good (≤2 mm), satisfactory (≤3 mm) or poor (>3mm). The identical procedure was performed on the preoperative lateral planning radiographs after first transposing the jig pin locations from the postoperative radiographs to these films, and second, tracing a 24 mm radius circle centered over the tibial intercondylar tubercles, or ICO. This circle represented the proposed IOL based on current recommendations of the ICO in relation to the long axis of the tibia [4]. The guide position, guide-to-osteotomy fit, and angle for the IOL was again determined, as well as the tibial tuberosity width.

The position of the proximal jig pin hole was determined on the postoperative radiographs in the proximodistal plane as the distance from the center of the jig pin hole to the proximal tibial joint surface, and in the craniocaudal plane as a percentage of the distance the pin was placed relative to the caudal tibial plateau (ratio of distance of the caudal tibial plateau to the pin:full length of the tibial plateau).

### Statistical analysis

Data were analyzed using SPSS for Windows, V22.0 (SPSS Inc., Chicago, IL; USA). The Shapiro-Wilk test was used to assess the normality of the data. Descriptive summaries were reported as means ± standard deviation (SD), and range for normally distributed data and medians, interquartile ranges (IQR), and total ranges for non-normally distributed data. For both the freehand osteotomy and the proposed IOL, the likelihood ratio chi square test was used to evaluate differences between saw guide positions and guide-to-osteotomy fit between Group 1 and 2, determine if a relationship existed between the guide-to-osteotomy fit and guide positions or jig pins located 3–4 mm distal to the stifle joint surface in Groups 1 and 2, and investigate the potential difference between saw guide positions and guide-to-osteotomy fit between the freehand osteotomy and proposed IOL. A Mann-Whitney U test was performed to evaluate differences in the jig arm angle between groups for the freehand osteotomy and proposed IOL. A Kruskal-Wallis test was performed to evaluate the relationship of jig arm angle to the guide-to-osteotomy fit for the freehand osteotomy and IOL groups. A one-way ANOVA was used to evaluate the relationship between the jig pin’s craniocaudal location on the tibia and the guide-to-osteotomy fit in the freehand osteotomy and IOL groups, and to evaluate the width of the tibial tuberosity in relation to the guide-to-osteotomy fit in the proposed IOL. A Student’s t-test was used to evaluate differences in craniocaudal proximal jig pin placement between Group 1 and 2. Statistical significance was set at *P* < 0.05.

## Results

The records of 130 dogs that underwent a TPLO surgery were reviewed for Group 1. Ninety-four cases were excluded, reasons included: no jig used (n = 57), saw blade radius other than 24 mm (n = 11), procedures performed by a resident (n = 8), and proximal jig pin hole not clearly identified on radiographs (n = 14) due to implant positioning, or obliquity of the tibia relative to the radiographic positioning. Immediate postoperative radiographs were missing for 4 cases. A total of 36 dogs (40 stifle joints) were further evaluated for appropriateness of the osteotomy position as per our definition using the individual orthogonal displacements to the DOE and minimal tibial tuberosity width, which resulted in the elimination of an additional 11 dogs (13 stifle joints). A total of 27 stifle joints, were included for analysis in Group 1. In this group, the mean DOE was 5.4 mm ±1.9 mm SD (range 0 mm to 9.6 mm) with a mean tibial tuberosity width of 12.6 mm ±2.1 mm SD (range 10 mm to 17 mm). The center of the cluster (CC) was located 3.7 mm ±2.1 mm SD (range 0 mm to 7 mm) caudal and distal by 3.0 mm ±2.3 mm SD (range 6.6 mm distal to 2.3 mm proximal) to the IOL.

Two hundred ninety-four records of dogs that underwent a TPLO procedure were reviewed for Group 2. Of those, 215 were excluded because they were performed either without an alignment jig, or using an alignment jig and saw guide. Among the remaining 79 TPLO procedures, cases were excluded for use of a saw blade radius other than 24 mm (n = 34) and difficulty identifying the proximal jig pin on the mediolateral radiograph (n = 5). Once again, a total of 38 dogs (40 stifle joints) were further evaluated for appropriateness of the osteotomy position as per our definition using the individual orthogonal displacements to the DOE and minimal tibial tuberosity width, which resulted in the elimination of an additional 3 dogs (3 stifle joints). A total of 35 dogs (37 stifle joints) were included for analysis in Group 2. In this group, the mean DOE was 4.0 mm ±1.7 mm SD (range 1 mm to 8.6 mm) with a mean tibial tuberosity width of 12.2 mm ±1.7 mm SD (range 10 mm to 16 mm). The center of the cluster (CC) was located caudal by 2.5 mm ±2.0 mm SD (range 1.4 mm cranial to 6.4 mm caudal) and distal by 1.8 mm ±2.3 mm SD (range 6.0 mm distal to 2.2 mm proximal) to the IOL.

Of the 12 possible saw guide positions, 9 combinations had the most accurate fit for all freehand osteotomies (Table 1). Within Groups 1 and 2, the most common saw guide positions were B2 and A2 (33.3% each) and B3 (62.1%), respectively. Four combinations had the most accurate fit for all proposed IOLs (Table 2). Within Group 1 and 2, the most common saw guide positions were B2 (61.5%) and B3 (59.5%), respectively. The difference in the position distribution between the two groups was significant for both the freehand osteotomy and proposed IOL (*P* < 0.001 and *P* = 0.022). There was no significant difference for saw guide positions between freehand osteotomy and proposed IOL (*P* = 0.190).

**Table 1:** The distribution of frequency for each saw guide position as the best guide-to-osteotomy fit for freehand osteotomies based on group.

Saw guide position	A1	A2	A3	A4	B1	B2	B3	B4	C3	Total
Group 1	2	9	4	0	0	9	3	0	0	27
Group 2	0	0	7	1	1	3	23	1	1	37
Total	2	9	11	1	1	12	26	1	1	64

**Table 2:** The distribution of frequency for each saw guide position as the best guide-to-osteotomy fit for the proposed ideal osteotomy location (IOL) based on group.

Saw guide position	A1	A2	A3	B1	B2	B3	C1	C2	C3	Total
Group 1	0	0	0	0	16	9	0	0	1	26*
Group 2	0	0	0	0	10	22	0	3	2	37
Total	0	0	0	0	26	31	0	3	3	63

* The preoperative radiograph was missing in one case.

Among the freehand TPLOs the median deviation of the proximal jig arm angle in Group 1 was +16° with a range of -20° to +30° (IQR -5° to +20°) from a baseline jig angle of 90°. In Group 2, the median angle was -5° with a range of -25° to +30° (IQR -12° to +12°). There was a significant difference between the jig arm angle in each group (*P* < 0.001), but no difference between jig arm angle on the guide-to-osteotomy fit (*P* = 0.665). In the proposed IOL cohort, the median deviation of the proximal jig arm angle in Group 1 was +9° with a range of -25° to +26° (IQR -4.5° to +18.5°). In Group 2, the median angle was +5° with a range of -25° to +30° (IQR +0.5° to +18.5°). There was no significant difference between the jig arm angle in each group nor between jig arm angle on the guide-to-osteotomy fit (*P* = 0.894 and *P* = 0.884, respectively).

Within Group 1 of the freehand osteotomies, 13 (48.1%) were considered to have excellent fits with the saw guide (≤1 mm offset), 8 (29.6%) had good fits (≤2 mm offset) and 5 (18.5%) had satisfactory fits (≤3 mm offset). Only 1 osteotomy (3.7%) deviated greater than 3 mm from all possible jig saw guide positions and was considered a poor fit. In Group 2, 23 (62.2%) had excellent fits, 11 (29.7%) had good fits, 3 (8.1%) had satisfactory fits, and no cases had poor fits. There was no significant difference between the two groups and no association between guide position and guide-to-osteotomy fit (*P* = 0.306 and *P* = 0.404, respectively).

Within Group 1 of the IOLs, 16 (61.5%) were considered to have excellent fits, 6 (23.1%) had good fits, and 4 (15.4%) had satisfactory fits (one preoperative radiograph was missing in group 1). In Group 2, 32 (86.5%) had excellent fits, 2 (5.4%) had good fits, and 3 (8.1%) had satisfactory fits. No proposed IOLs in Group 1 or 2 had poor fits. There was no significant difference between the two groups (*P* = 0.057). There was no association between guide position and guide-to-osteotomy fit (*P* = 0.090). There was no difference between guide to osteotomy fit for the freehand osteotomies and IOLs (*P* = 0.373)

In Groups 1 and 2, the proximal jig pin locations were a median of 4 mm and 5 mm distal to the joint surface with ranges of 1 mm to 9 mm (IQR 3 mm to 5 mm) and 3 mm to 11 mm (IQR 4 mm to 7 mm), respectively. The number of cases with jig pins 3–4 mm distal to the plateau was not significantly different between the groups (*P* = 0.585). Of the 33 jig pins placed 3–4 mm distal to the plateau, 24 (72.7%) produced excellent fits and all produced satisfactory or better saw guide-to-osteotomy fits. The relationship between jig pins that were 3–4 mm distal to the plateau surface and the guide-to-osteotomy was statistically significant in both the freehand osteotomy and proposed IOL (*P* = 0.032 and *P* = 0.038, respectively).

In Groups 1 and 2, the jig pins were placed at a mean location of 39.5% (±12.1% SD, range 17% to 63%) and 27.9% (±6.7% SD, range 16% to 42%), respectively, as a percentage location along the tibial plateau from the caudal border. The difference between jig pin locations in a craniocaudal plane along the tibial plateau was statistically significant between Group 1 and Group 2 (*P* < 0.001). The location was also significantly related to guide-to-osteotomy fit for freehand osteotomies but not for proposed IOLs (*P* = 0.047 and *P* = 0.242, respectively).

The mean maximal tibial tuberosity width measured on the preoperative radiographs based on the proposed IOL was 12.8 mm (±3.3 mm SD, range 8 mm to 22 mm). Ten cases had tibial tuberosity widths less than 10 mm. There was no significant difference between the tibial tuberosity width and the guide to osteotomy fit (*P* = 0.307).

## Discussion

**Fig 4:**
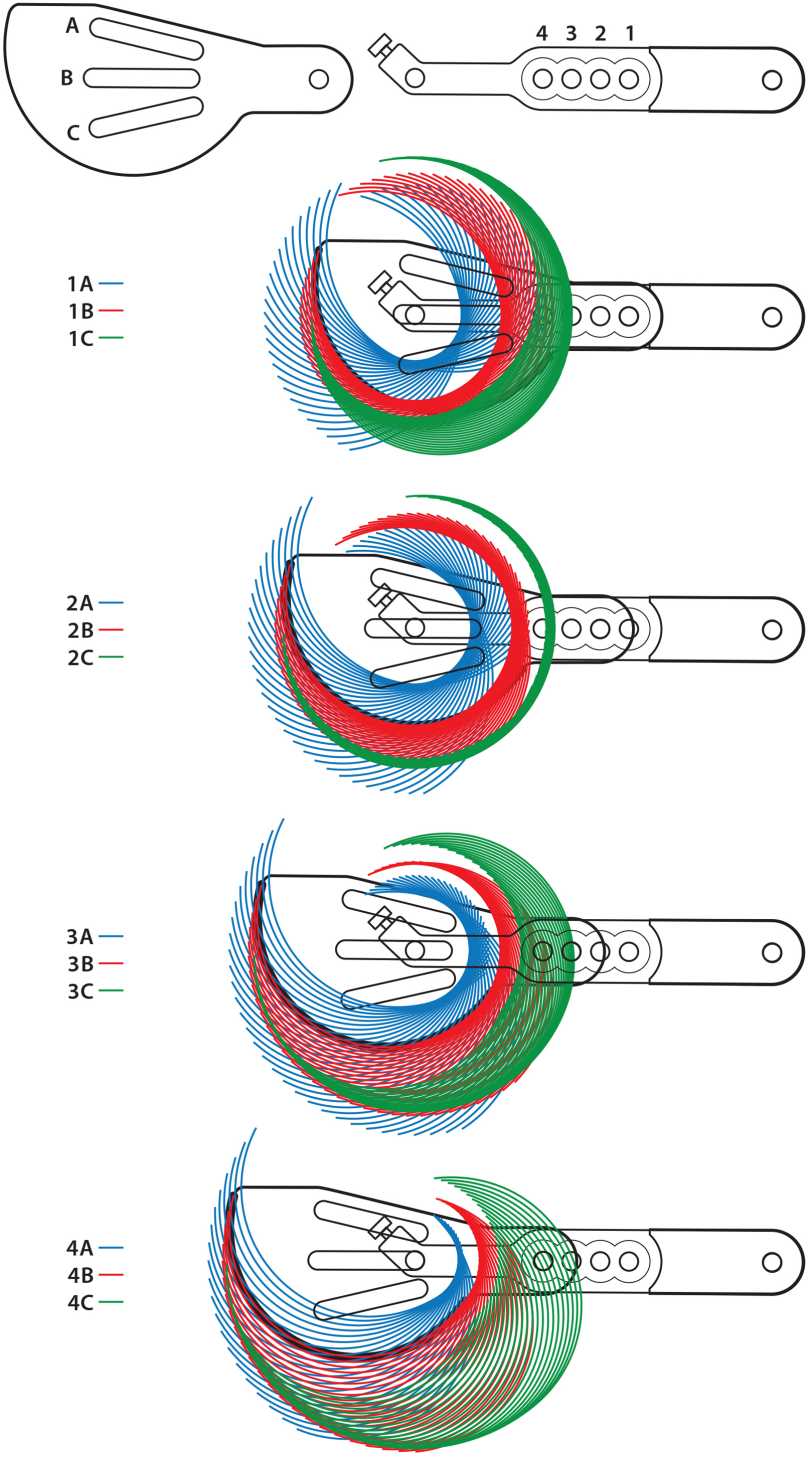
Depiction of the osteotomy options available with each of the twelve positions and ±30° from a baseline of 90° angulation of the proximal jig arm. Each shaded area is a result of angulating the jig arms in each of the positions selected (ABC/1234).

Overall, 98.4% of the clinical freehand TPLO and 100% of the proposed IOL positions were satisfactorily reproducible with the saw guide. With the 12 possible positions for attachment to the jig and the extensive spectrum of angles achievable with adjustment of the jig arm, the saw guide provides versatility in the range of positions and tibial coverage choices available to the surgeon. Despite the wide range of options, there are still small gaps in the coverage possible with the guide (Fig 4). Our data showed a wide variability in jig arm angles for the clinical cases, but this was not found to be a significant contributor to the guide-to-osteotomy fit.

**Fig 5:**
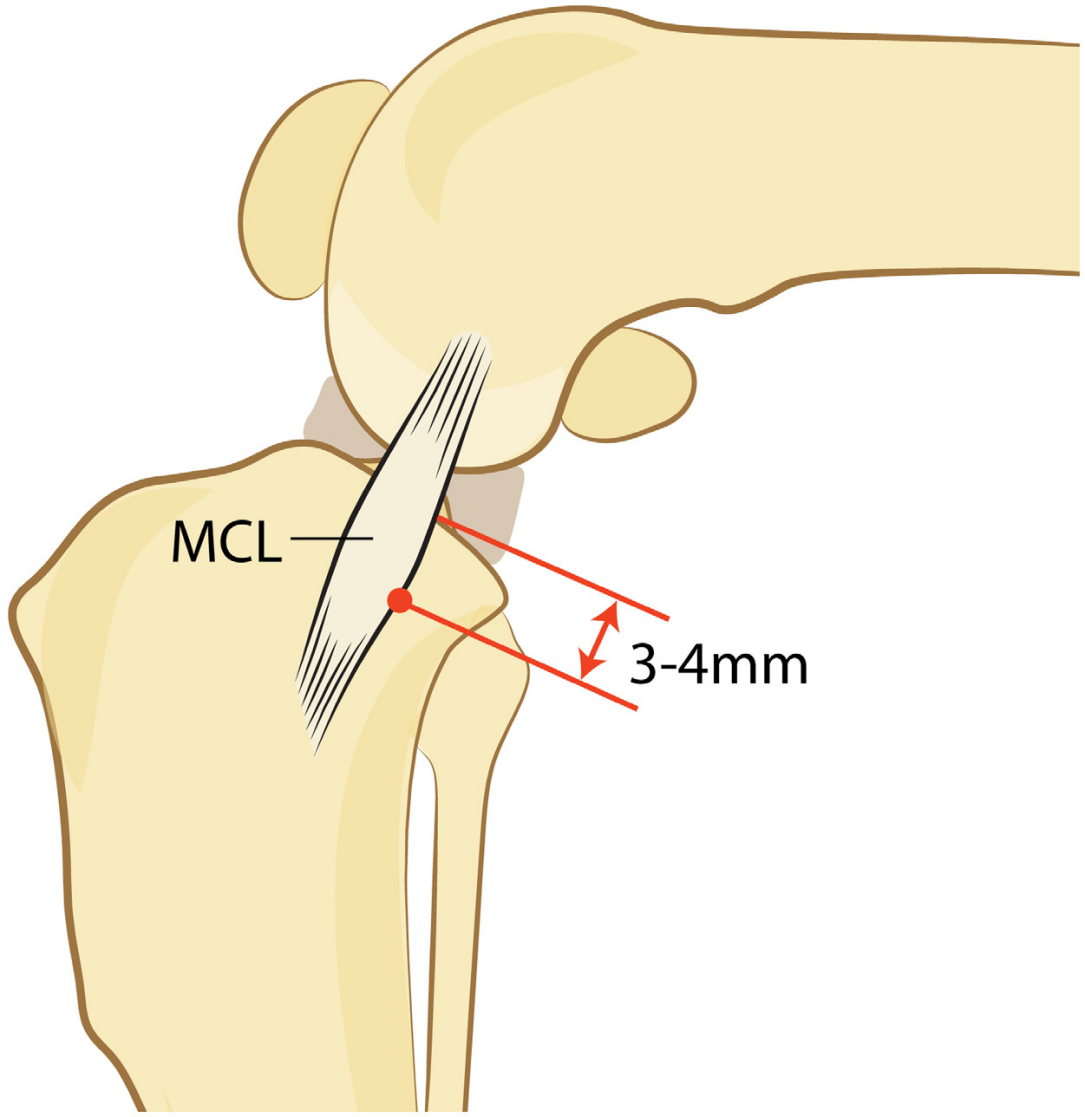
The proximal jig pin is inserted (dot) parallel to the joint surface and perpendicular to the tibial long axis, 3–4 mm distal to the joint and just caudal to the medial collateral ligament (MCL).

Accurate placement of the proximal jig pin parallel to the joint surface and perpendicular to the tibial long axis is paramount for successful use of the saw guide, as the saw guide physically attaches to the proximal jig pin and orients the osteotomy in direct relation to this pin. Current recommendations for the use of the alignment jig/saw guide include: the proximal-most aspect of the tibia identified via insertion of a 25-gauge needle immediately caudal to the MCL; insertion of a 3 mm jig pin at the caudal margin of the MCL and 3–4 mm distal to the needle position (Fig 5) [7,9]. When the procedure was first described, recommendations for placement of the proximal jig pin differed most notably with regards to the MCL; this position was through the MCL (Slocum B. Tibial plateau leveling osteotomy course. Eugene, OR, Slocum Enterprises Inc. 1998) [1]. One of the goals of this paper was to evaluate the effect of proximal jig pin location on the ability of the guide to match the clinical osteotomies; as such, two populations were recruited to represent the different positions relative to the MCL that followed the recommendations at the respective time periods: through the body of the MCL (Group 1) versus adjacent to the caudal margin of the MCL (Group 2). The recommended location of the proximal jig pin in a proximodistal direction from the joint surface has not been substantially altered since the inception of the TPLO.

Among the cases reviewed in this study, just over half had jig pins placed at the recommended distance of 3–4 mm from the tibial joint surface, with no difference found between the two groups. Placement 3–4 mm from the joint was statistically related to the guide-to-osteotomy fit. Evaluating the location of the jig pins in relation to the MCL was more problematic. Although the MCL is easily identifiable in surgery, it is impossible to discern its exact location on radiographs. In Group 2, the mean location of the pin in a craniocaudal orientation on the tibia was significantly more caudal than that of Group 1, as was anticipated, and the location on a craniocaudal plane of the tibia was found to be significantly associated with guide-to-osteotomy fit. Furthermore, a significant difference was seen in the preferred guide position between the two groups in both the freehand osteotomies and proposed IOLs, with Group 1 having more caudally placed guide-to-osteotomy fit positions, A2 and B2, to compensate the more cranial jig pin location, compared to B3 in Group 2 (Tables 1 and 2, Fig 4).

Using the mean location from Group 2 plus one standard deviation on each side gives a reasonable, conservative estimate of the ideal location of the jig pin for an average dog in a craniocaudal plane on the proximal tibia based on current recommendations, at 34.6% to 21.2%. This is further supported by a study investigating the anatomy of the canine MCL, in which radiographic markers were placed on the cranial and caudal borders of the MCL. Stifle radiographs were taken at 120° and 60° of flexion/extension. The locations of the caudal aspect of the MCL were 37.5% and 22.5%, respectively [11]. Extrapolating from this data, at 90° flexion, the position of the postoperative radiographs, we reasonably estimate the location to be midway between these distances, approximately at the caudal 1/3 of the tibial plateau. This position, without violating the MCL, ensures a more caudal and consistent placement of the proximal jig pin and allows more room for the plate head footprint, which also ensures a wider segment of bone between the tibial tuberosity and the osteotomy cranially. Placing a jig pin too far caudal is possible, although unlikely if placed as described adjacent to the caudal border of the MCL [7,9]. Caudal placement beyond this area will result in the pin penetrating the medial cortex of the tibia and exiting in the concave popliteal notch. The jig pin location in both the proximodistal and craniocaudal planes was significantly associated with the guide-to-osteotomy fit, supporting that the current pin placement recommendations work well for use with the saw guide.

The one freehand osteotomy that was not reproducible with the guide (poor fit), had a severely malpositioned proximal jig pin. The jig pin was located at 7 mm from the joint surface and 36% the length of the tibial plateau from caudal. This underscores the importance of appropriate jig pin placement when using the alignment jig/saw guide.

For novice surgeons, initiation of a precise freehand osteotomy has proven difficult since it requires proper placement and orientation of the saw blade on a sloped bone surface in both the frontal and sagittal planes, which results in the saw blade “skidding” along the cortex when the cut is initiated. The latter is a result of the curved saw blade only in contact with the bone at its caudal-most aspect. A common technique involves starting the osteotomy freehand requires that the saw be angled initially so as to ensure contact of the entire blade on the sloped bone surface, and then continued in the appropriate perpendicular orientation to the sagittal and frontal planes only after the initial kerf has been established. The saw guide facilitates the initiation of the osteotomy by providing a matching curved surface on which to brace the saw. This technique is successful in eliminating the saw’s “skid” across the medial cortex as long as the saw is kept in contact with the saw guide [8]. Additionally, an osteotomy made using the saw guide is more accurate with regards to consistency with preoperative planning and appropriate centering of the osteotomy [8,9]. The most common error in osteotomy placement is a caudal and distal positioning [9]. One hypothesis is the inadvertent tendency to center the osteotomy over the proximal jig pin [9]. Because the guide incorporates the jig pin into the osteotomy planning in a consistent manner, this error is avoided, provided the jig pin itself is properly located.

The proposed IOL was drawn on preoperative radiographs to evaluate the saw guide’s ability to not only reproduce appropriate freehand osteotomy locations in clinical cases, but also to determine if the jig pin positions could also replicate the current recommendation of centering the osteotomy over the intercondylar tubercles at the ICO [4]. All proposed IOL were satisfactorily reproducible, despite the range in proximal jig pin locations. However, 10 proposed IOL positions had maximal tibial tuberosity widths of <10 mm, a risk factor for tibial tuberosity fracture [3,6]. This suggest that these cases may have been better suited with a smaller radius saw blade.

The saw guide is simple to apply and can be used as a learning tool for inexperienced surgeons. The guide can obtain the same positions as those obtained by experienced surgeons in the appropriate locations; the latter defined by our assessment of DOE and tibial tuberosity width. An additional benefit of the saw guide is as a visual aid prior to performing the osteotomy. Adjusting the guide over the top of a TPLO plate placed on the bone, as determined by the anatomic landmarks, or based on preoperative planning until the desired plate coverage and footprint is obtained, confirms the osteotomy location before cutting the bone. Placement of the guide over the proposed osteotomy site also allows for intraoperative assessment of tibial tuberosity width, again, before commencing the osteotomy. Application of the guide is quick and easy, adding only a few seconds to a procedure already using an alignment jig. Intraoperative time may actually be decreased because the difficult step of initiating the cut is expedited and simplified, especially in the hands of less experienced surgeons; however, this assumption remains to be proven.

As with all retrospective studies, certain limitations are inevitable. Preplanning was performed by two different methods, either by transposing measurements made from the preoperative radiographs or assessing the footprint of the plate intraoperatively. Our experience has shown that the osteotomy locations are comparable regardless of the method used; however, this assessment remains to be documented. Regardless, both methods are in widespread use clinically. Certainly, the comparison to a freehand osteotomy, no matter the plan to place the osteotomy, and despite being performed by experienced surgeons, cannot validate that the osteotomy performed was in the ideal position, just that the alignment jig/saw guide could replicate this position. As such, we attempted to define an appropriate TPLO based upon the recommendations of a centered osteotomy and a minimal tibial tuberosity width [3,4,6]. Two previous clinical studies in over 600 clinical cases have been described whereby the accuracy of a TPLO osteotomy with these guidelines was demonstrated [6,9]. In these latter studies, the ACO was compared to the ICO. The results showed in one a DOE of 1.13 mm mean ±3.21 mm SD (freehand with footprint assessment only) and 0.48 mm mean ±2.28 mm SD (preplanned position), and in the other two methods of preplanning, with a DOE of 5.6 mm mean ±2.5 mm SD (freehand) and 3.4 mm mean ±1.8 mm SD (jig and saw guide), respectively [6,9]. Based upon these reported positions, we elected to define an appropriate osteotomy performed by experienced surgeons to have a DOE within this range based on a ≤7 mm displacement from the centers in either x (craniocaudal) or y (proximodistal) positioning, and a minimal tibial tuberosity width of 10. More accurate positioning closer to the ICO resulted in fewer postoperative complications [6]. Positioning of the actual osteotomy was found to be more distal and caudal to the ICO in most instances [6,9]. The latter is recognized to occur so as to maximize tibial tuberosity width and also ensure sufficient room for the footprint of the head of the plate [9]. Our findings are consistent with these observations.

Another limitation is the radiographic analysis of the proximal jig pin location. The distance of the jig pin hole from the proximal tibial plateau can be readily identified and reliably measured on lateral postoperative radiographs, but the location of the jig pin in relation to the caudal aspect of the MCL is less exact. There are significant limitations to this estimation, such as anatomic variations innate to each animal and in cases with osteoarthritis, where osteophytosis cranially and caudally on the tibial plateau can obscure these landmarks. The location of the MCL on the tibial plateau also changes based on the degree of stifle flexion/extension [11,12]. All of these factors could also have contributed to the wide variation in jig pin placement reported.

Currently, the saw guide is offered with radii of 24 mm, 27 mm, and 30 mm. Although the osteotomy size evaluated was limited to a single radius of 24 mm in this study, it is reasonable to assume that a similar ability to reproduce well-positioned osteotomies exists for the 27 mm and 30 mm saw guides. Support for this statement have been previously reported [8,9].

Based on our results, we propose that the alignment jig/saw guide can reliably be used to replicate an appropriate TPLO osteotomy performed freehand by an experienced surgeon and accommodate an accurate centering of an IOL closer to the ICO, although dependent on appropriate proximal jig pin placement. Given the ease of use, visual cues provided by the saw guide, and the versatility of positioning, the saw guide may be a helpful tool for novice surgeons. It would likely be a useful and helpful tool in a clinical/teaching role for those individuals first learning to successfully perform the technique, and result in better consistency of both osteotomy and subsequent plate position. We would also surmise that a similar benefit could be attained if also utilized by experienced surgeons.

## Supporting Information

S1 File TPLO Technique Guide (DePuy Synthes^®^ Vet, West Chester, PA; USA). (PDF)

S2 File DePuy Synthes Fig 3 permission letter. (PDF)

S3 File Revised DePuy Synthes Fig 3 permission request letter. (PDF)
