# Supplementary material for: Novel TPLO Alignment Jig/Saw Guide Reproduces Freehand and Ideal Osteotomy Positions
Source: PLoS One. 2016 Aug 24;11(8):e0161110. doi: 10.1371/journal.pone.0161110 (PMC4996453; doi:10.1371/journal.pone.0161110)
Supplement: S1 File — (PDF) [file pone.0161110.s001.pdf]

DePuy Synthes VET

COMPANIES OF Johnson & Johnson

STANDARD TIBIAL PLATEAU LEVELING OSTEOTOMY (TPLO) SYSTEM

For stabilizing osteotomies of the canine proximal tibia

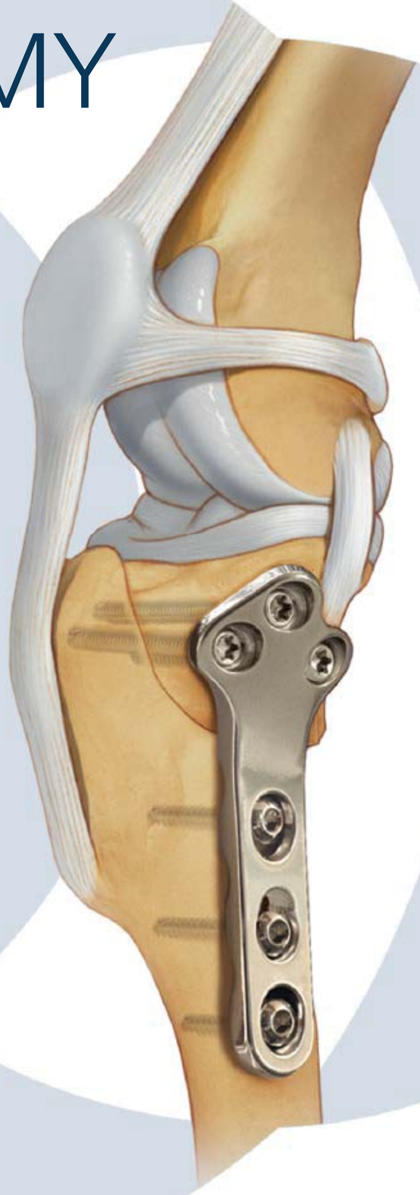

SURGICAL TECHNIQUE

TABLE OF CONTENTS

INTRODUCTION	Standard Tibial Plateau Leveling Osteotomy System	2
	TPLO Saw Blades	6
	AO Principles	7
	Indications	7
	Clinical Cases	8
SURGICAL TECHNIQUE	Plate Contouring and Positioning	11
	Drill Guide Technique	12
	Jig Pin/Saw Guide Technique	14
	Screw Insertion Sequence	19
PRODUCT INFORMATION	Implants	22
	Instruments for Locking Screws	26
	Instruments	29
	Set Information	32
	TPLO Rotation Quick Reference Chart	34

STANDARD TIBIAL PLATEAU LEVELING OSTEOTOMY (TPLO) SYSTEM

For stabilizing osteotomies of the canine proximal tibia

The DePuy Synthes Tibial Plateau Leveling Osteotomy (TPLO) plate is part of a stainless steel plate and screw system that merges locking screw technology with conventional plating techniques. The TPLO plate has many similarities to existing bone fixation plates, with a few important improvements. The technical innovation of locking screws and an anatomical contour provide the ability to create a fixed-angle construct while following familiar AO plating principles.

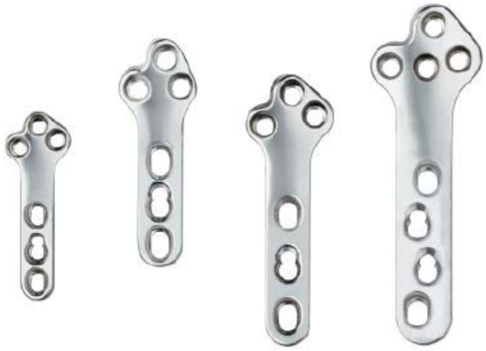

The standard system includes the 2.7 mm, 3.5 mm small, 3.5 mm, and 3.5 mm broad plates. The mini TPLO system technique guide will cover the 2.0 mm and 2.4 mm TPLO plates.

Features
- Available in 2.7 mm, 3.5 mm small, 3.5 mm, and 3.5 mm broad
- Available in right and left versions
- Uses either conventional or locking screws
- Precontoured for anatomic fit
- Plate head specifically designed to engage more bone
- Screw trajectory in head holes is designed to minimize likelihood of penetrating articular surface and osteotomy

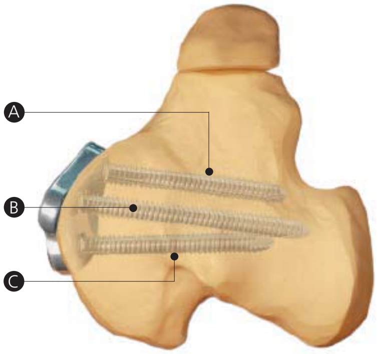

Head screw angulations

A. Cranial screw: 5° caudal
B. Proximal screw: 3° distal/5° caudal
C. Caudal screw: 3° cranial

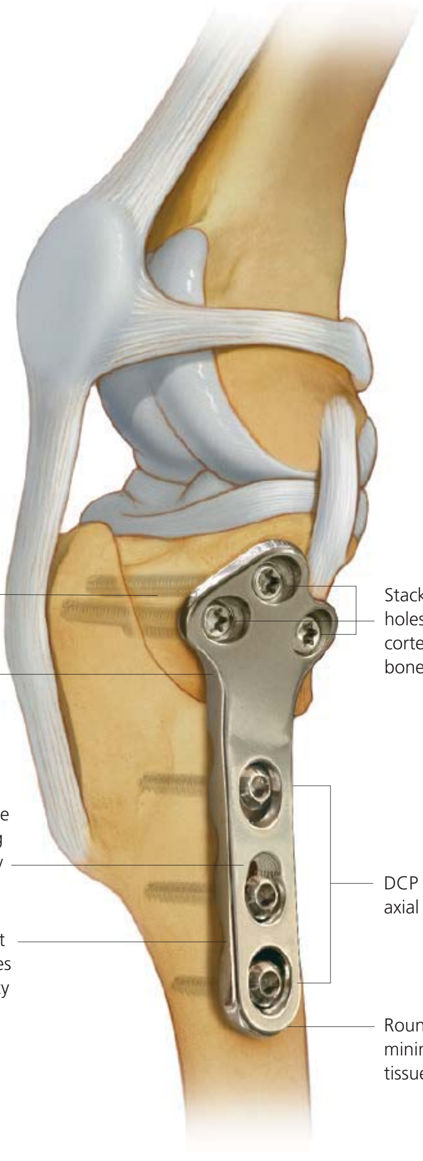

Standard TPLO Jig

- Multiple saw guide positions allow for versatile positioning to accommodate a broad spectrum of patient anatomies
- Maintains stability and limb alignment
- Vibration-resistant hinge screws eliminate loosening and are easily tightened by hand
- Hardened steel jig pin screws resist stripping
- Attaches to bone with a 3.0 mm pin
- Easy disassembly for removal and cleaning
- Works with DePuy Synthes saw guides
- Jig arm design allows a longer set screw which lessens the chance of accidental loss
- Jig hole is clear once any threads are observed as the jig pin screw is loosened

Saw Guides for Standard TPLO Jig

- Facilitates saw positioning for the osteotomy
- Stabilizes and guides the saw
- Eliminates chatter and walking of the saw blade
- Available in three different radii: 24 mm, 27 mm, and 30 mm
- Designed for use with DePuy Synthes crescentic saw blades
- Vibration-resistant saw guide screw eliminates loosening and is easily tightened by hand

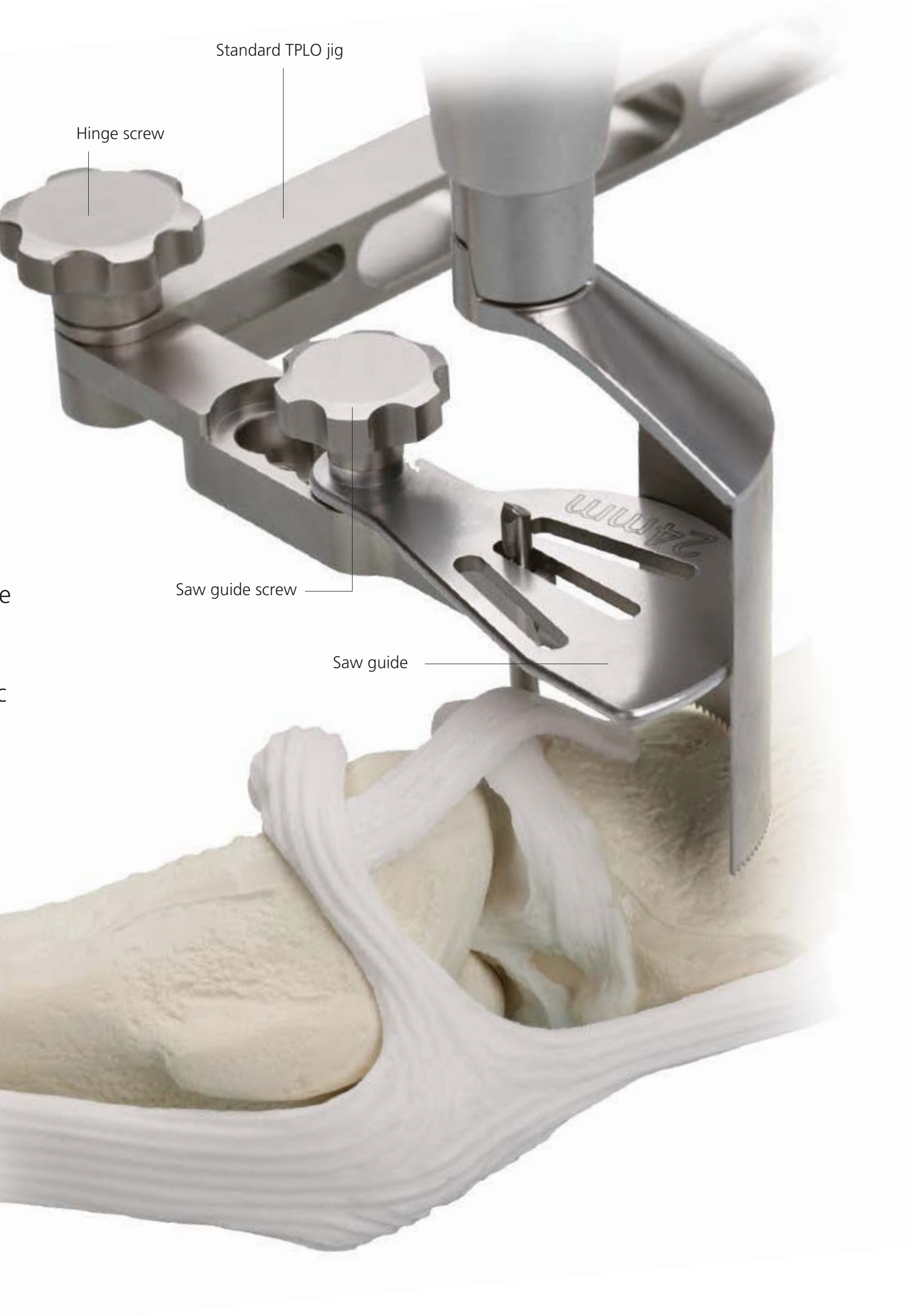

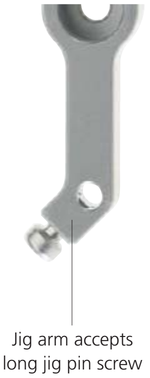

Holes

The DePuy Synthes Vet TPLO plate is designed with three distinct screw-hole technologies to accommodate all plating modalities. Along its shaft are two dynamic compression plate (DCP) holes separated by one or two central locking compression plate (LCP) Combi holes; in its head are three or four stacked Combi holes.

The DCP holes accept cortex screws that may be placed in either loaded or neutral positions, depending on whether or not interfragmentary compression is desired (see Universal Drill Guide for more detail).

The LCP Combi hole(s) in the center of the plate shaft accepts either cortex screws or locking screws. The cortex screw should be placed in the unthreaded portion of the locking Combi hole in either a loaded or neutral position. Alternatively, a locking screw may be used in the threaded portion of the Combi hole when indicated.

The three stacked Combi holes in the plate head accept either cortex, cancellous bone, or locking screws. If locking screws are to be used in conjunction with cortex or cancellous bone screws in the plate head, the cortex screws must be inserted and tightened first, before any locking screws are inserted. If cortex screws are used, the plate must be appropriately contoured to the bone.

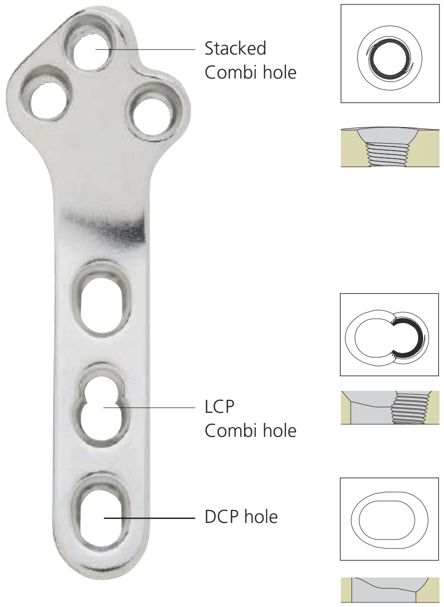

Fixed-Angle Stability

The threads on the head of the locking screws lock into the threaded plate holes to form a fixed-angle construct that will increase load transfer between the plate and bone. When compared to conventional plate-and-screw constructs, the angular and axial stability of locking screws increases the strength of the construct under load without requiring precise anatomical contouring.

Angled threaded holes in the head of the TPLO plate help ensure that screws are angled away from the articular surface.

Anatomical Contour

The anatomically shaped TPLO plate is contoured to match the medial aspect of the canine proximal tibia. This can reduce or eliminate the need for plate contouring.

Limited Contact

The limited-contact shaft design reduces plate-to-bone contact area to help preserve vascularity and to support bone healing.

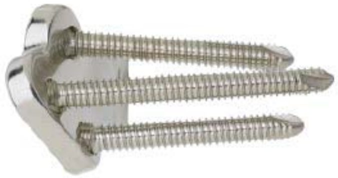

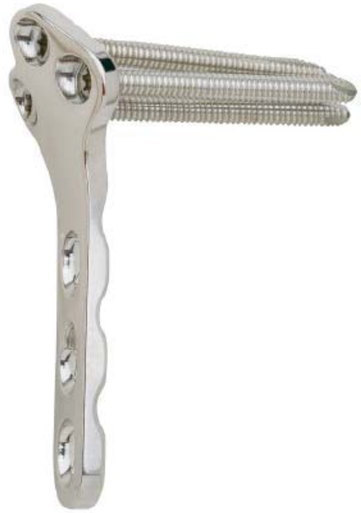

All implants are made of implant-quality 316L stainless steel.

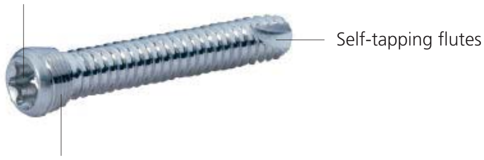

Screwhead
The tapered, double-lead machine thread on the head of the locking screw engages the threads of the locking holes. The resulting fixed-angle construct provides stable fixation of the bone fragments without having to compress the plate to the bone. A perfectly contoured plate is therefore not required to achieve fixation and maintain proper alignment.

Thread Profile
Because locking screws do not compress the plate to the bone, the "pull-out" mode of failure is not applicable to locking screws. For this reason, locking screws are made with a smaller thread profile and a larger core diameter. This results in increased mechanical strength over comparably sized cortex and cancellous bone screws.

Note: The locking screws are self-tapping.

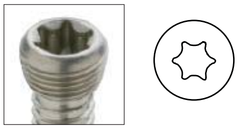

Drive Mechanism
The StarDrive recess of a locking screw provides three significant improvements over an internal hex drive. First, "stripping" of the screwhead is minimized as a failure mode, which results in a much higher tolerance to wear for the screwdriver. Second, the tapered StarDrive recess provides automatic screw retention without the need for an additional screw holding mechanism. Third, the more efficient StarDrive recess allows a smaller screwhead and allow the screwhead to sit flush with the plate.

All implants are made of implant-quality 316L stainless steel.

TPLO SAW BLADES

Saw Attachment and Saw Blades for TPLO

- Specially designed saw attachment for tibial plateau leveling osteotomies
- Seven saw blades, with radii ranging from 12 mm–30 mm
- Simple and stable connection of the saw blade into the saw attachment
- Thin saw blades offer excellent cutting performance and minimal bone removal (cutting thickness is 0.6 mm)

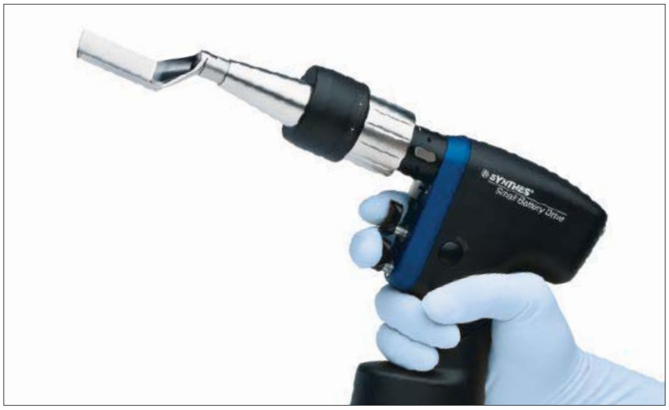

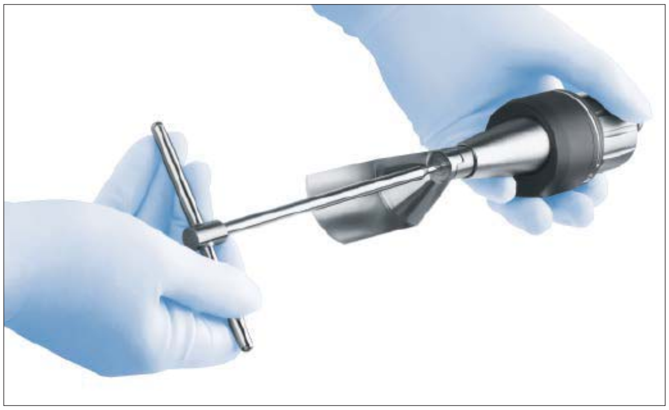

AO PRINCIPLES

In 1958, the AO formulated four basic principles, which have become the guidelines for internal fixation.[1] They are:

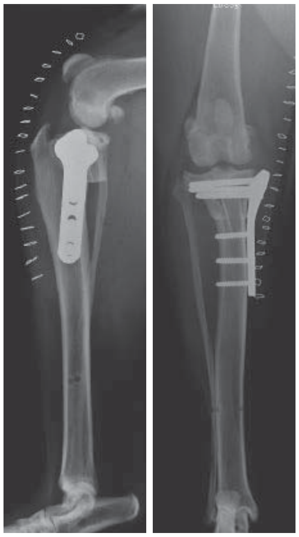

Anatomic reduction
Fracture reduction and fixation to restore anatomical relationships.

Stable fixation
Stability by fixation or splintage, as the personality of the fracture and the injury requires.

Preservation of blood supply
Preservation of the blood supply to soft tissue and bone by careful handling and gentle reduction techniques.

Early, active mobilization
Early, safe mobilization of the part and patient.

INDICATIONS

The DePuy Synthes Tibial Plateau Leveling Osteotomy (TPLO) System is intended for use in stabilizing osteotomies of the canine proximal tibia.

1. Johnson AL, Houlton J, Vannini R. *AO Principles of Fracture Management in the Dog and Cat.* Stuttgart: Thieme; 2005.

CLINICAL CASES

Case 1: 3.5 mm TPLO Plate

A 74-lb., six-year-old, neutered female Labrador presented with acute lameness and a painful stifle.

The use of 3 locking screws in the 3.5 mm plate head ensures optimal stabilization of the proximal portion of the tibia. The distal portion of the tibia is stabilized using cortex screws in the plate shaft.

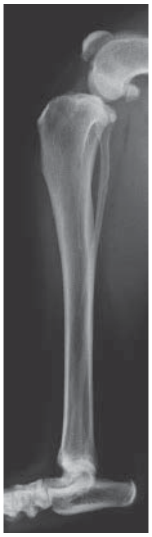
Preoperative Lateral

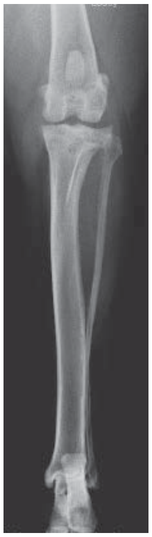
Preoperative Cr-Ca

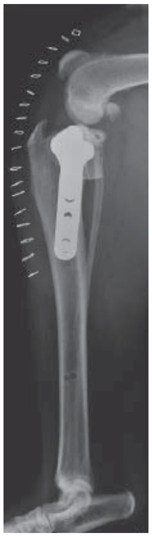
Postoperative Lateral

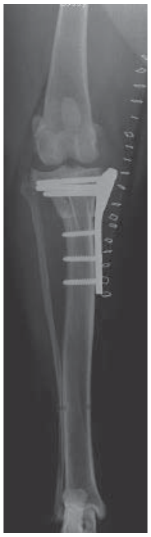
Postoperative Cr-Ca

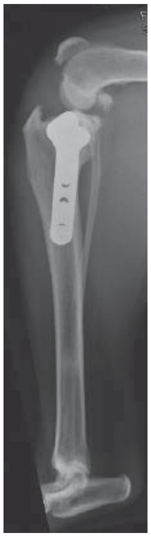
8-week Follow-up Lateral

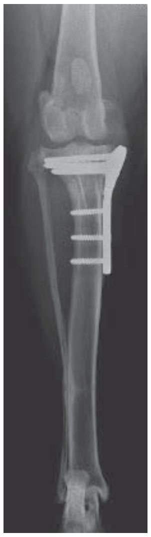
8-week Follow-up Cr-Ca

Case 2: 3.5 mm TPLO Plate

An 86-lb., three-year-old, neutered female German Shepherd presented with left hind limb lameness of 1-week duration.

This patient was previously diagnosed with multicentric B-cell lymphoma. The medication regime included Leukeran 30 mg every 14 days, methotrexate 2.5 mg twice weekly, prednisone 25 mg every other day, and Pepcid 20 mg once daily.

Similarly to Case 1, 3 locking screws were used in the 3.5 mm plate head to ensure optimal stabilization of the proximal portion of the tibia. The distal portion of the tibia was stabilized using cortex screws in the plate shaft.

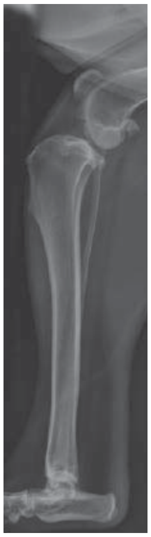
Preoperative Lateral

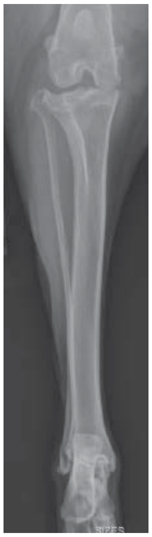
Preoperative Cr-Ca

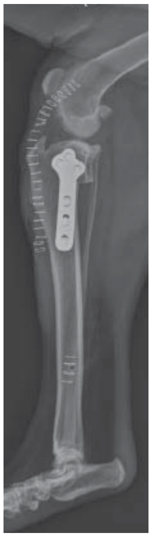
Postoperative Lateral

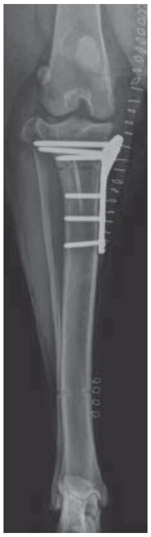
Postoperative Cr-Ca

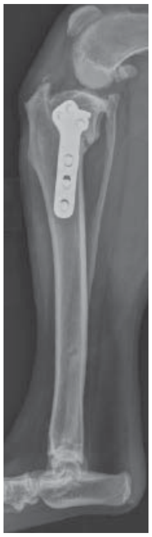
8-week Follow-up Lateral

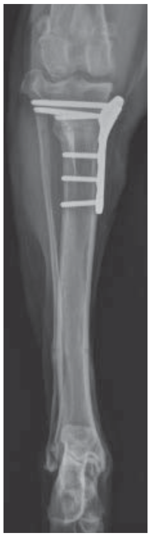
8-week Follow-up Cr-Ca

Case 3: 2.7 mm TPLO Plate

A 48-lb., 8-year-old, Cocker Spaniel presented with right hind leg lameness as well as medial patellar luxation (MPL). The MPL was repaired with trochlear block recession, medial retinacular release, lateral imbrication, and lateral displacement of the tibial tuberosity during the TPLO.

The TPLO was performed using 3 locking screws in the 2.7 mm plate head to ensure optimal stabilization of the proximal portion of the tibia. The distal portion of the tibia was stabilized using cortex screws in the plate shaft.

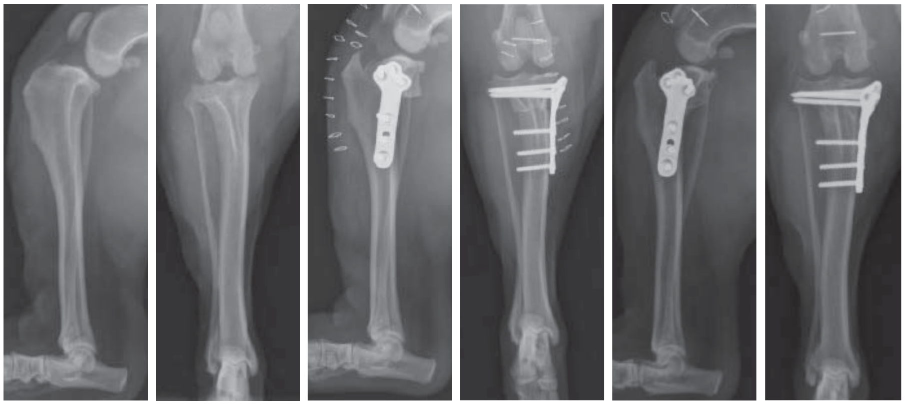

Preoperative Lateral | Preoperative Cr-Ca | Postoperative Lateral | Postoperative Cr-Ca | 8-week Follow-up Lateral | 8-week Follow-up Cr-Ca

Case 4: 3.5 mm Small TPLO Plate

A 45-lb., 12-year-old, neutered male Brittany Spaniel presented with acute lameness after jumping off a deck.

A 3.5 mm small TPLO plate was used, with 3 locking screws in the head of the plate, and 3 cortex screws in the shaft.

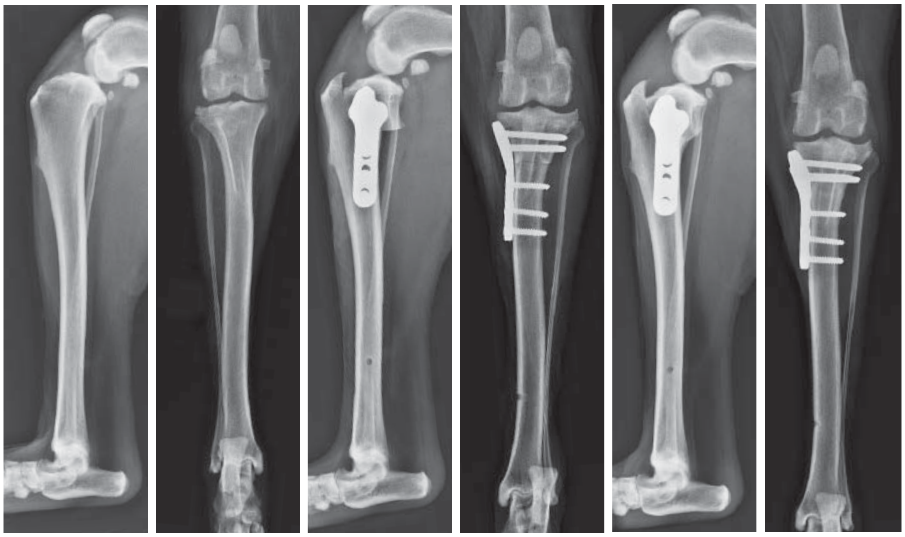

Preoperative Lateral | Preoperative Cr-Ca | Postoperative Lateral | Postoperative Cr-Ca | 8-week Follow-up Lateral | 8-week Follow-up Cr-Ca

Case 5: 3.5 mm Broad TPLO Plate

A 93-lb., 3-year-old, spayed female Mastiff presented with intermittent right hind lameness that did not resolve despite anti-inflammatory medications and exercise restriction, and was occasionally lame on the left hind leg after increased activity.

Radiographs of both stifles showed soft tissue swelling, effusion, and bony changes around both stifle joints consistent with bilateral cruciate tears. A TPLO was performed first on her right stifle, then shortly after on her left stifle.

Four locking screws were used in the 3.5 mm broad plate head to ensure optimal stabilization of the proximal portion of the tibia. The distal portion of the tibia was stabilized using cortex screws in the plate shaft.

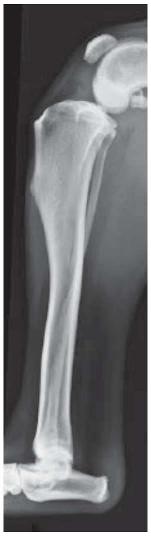

Preoperative Lateral

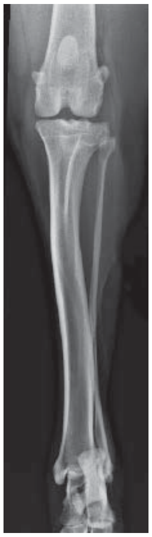

Preoperative Cr-Ca

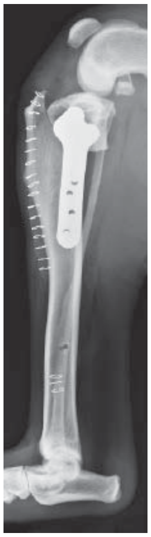

Postoperative Lateral

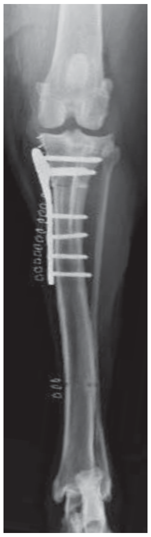

Postoperative Cr-Ca

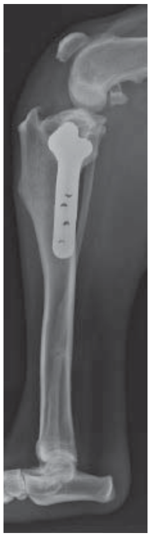

8-week Follow-up Lateral

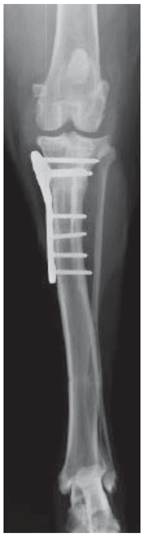

8-week Follow-up Cr-Ca

Plate Contouring

If only locking screws are used in the plate head, contouring of the plate is generally not required. The distal section must be pressed firmly to the diaphysis as standard screws are used to secure this position of plate. Some plate contouring to the shaft may be necessary to ensure the plate is flush with the bone.

If conventional screws (cortex or cancellous) are used in the plate head, the following precautions are necessary:

1. Because conventional screws pull the bone to the plate, contouring of the plate may be required to ensure plate contact with the bone.
2. If conventional screws are used in combination with locking screws, conventional screws must be inserted and fully tightened prior to inserting locking screw(s).

Note: Contouring of the plate will redirect the angle of the locking screws. It is best to avoid contouring around the head holes as this can distort the internal threads.

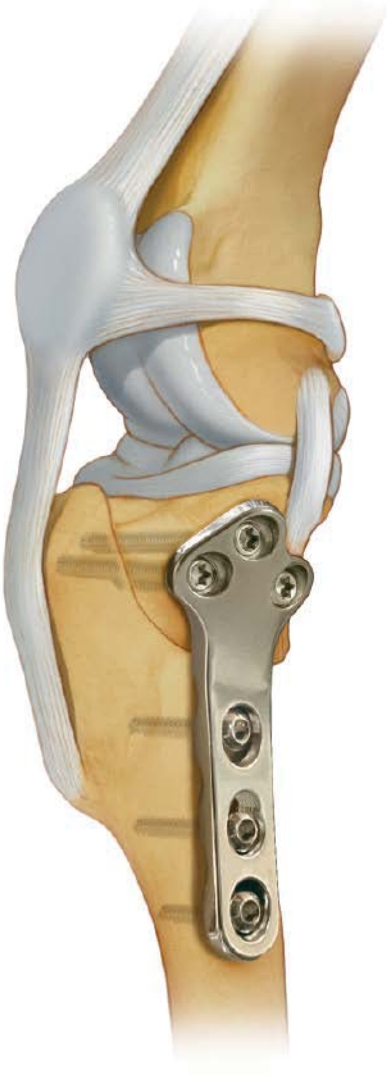

Plate Positioning

The TPLO plate should be positioned on the medial surface of the tibia, in a manner that best fits the bone contour and osteotomy. The plate is designed to be placed very proximally, just distal to the articular surface. The proximal head screw is angled 3° distal/5° caudal, away from the articular surface.

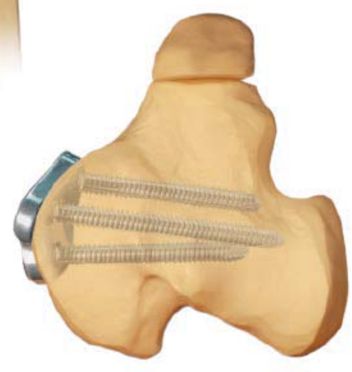

DRILL GUIDE TECHNIQUE

Threaded Drill Guide

Instruments	
312.648	2.8 mm Threaded Drill Guide, for 3.5 mm locking screws
313.353	2.0 mm LCP Solid Threaded Drill Guide, for 2.7 mm locking screws

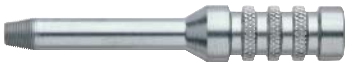

312.648

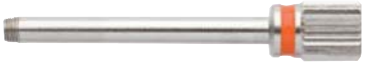

313.353

When a locking screw is placed, a threaded drill guide **must** be used for guiding the drill bit in the proper direction.

Note: The threaded drill guide can also be used intraoperatively as a reference for visualizing the angle at which the locking screws will be directed into the bone.

The 2.0 mm threaded drill guides fit the threaded holes of the 2.7 mm TPLO plates. The 2.8 mm threaded drill guides fit the threaded holes of the 3.5 mm TPLO Plates.

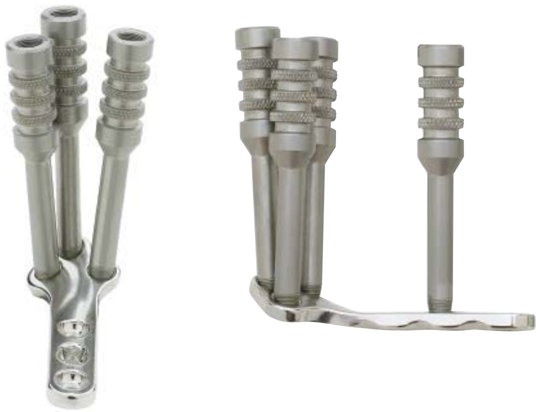

Universal Drill Guide

**Instruments**	
323.26	2.7 mm Universal Drill Guide
323.36	3.5 mm Universal Drill Guide

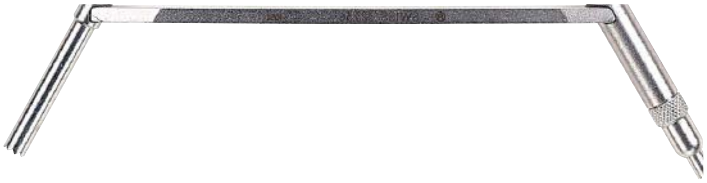
323.26

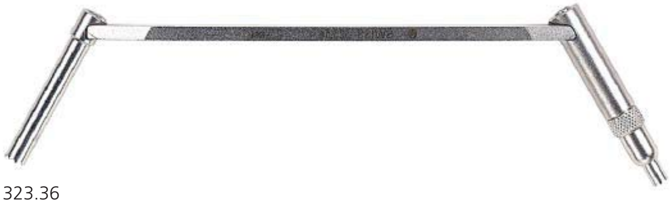
323.36

The universal drill guide is used to place conventional screws in a neutral position or load position. If the screw is intended to achieve interfragmentary compression, the universal drill guide should be placed in the load position, as shown and described in the figure to the right. If the screw is not used to provide interfragmentary compression, the universal drill guide should be placed in the neutral position.

Compression (load) Position
Compression is achieved by placing the universal drill guide in the eccentric position, and maintaining the drill guide body above the plate as shown.

Neutral Position
Neutral position is achieved by placing the universal drill guide in the eccentric position, then compressing the drill guide body into the hole, which will shift the drill guide into the neutral position as shown.

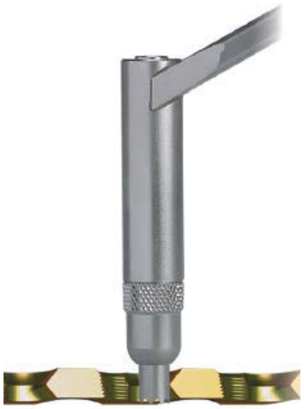
Compression (load)

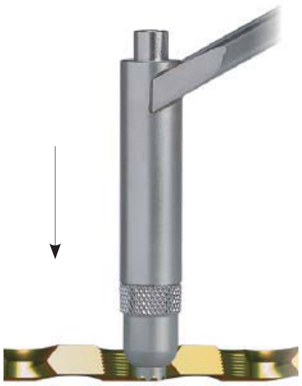
Neutral

JIG PIN/SAW GUIDE TECHNIQUE

1

Insert Proximal Jig Pin

Instruments

VQ0001.00	Standard TPLO Jig for use with 24 mm, 27 mm, 30 mm Saw Guides
VW3001.15	3.0 mm Kirschner Wire, 150 mm (jig pin)

Make a standard medial approach to the proximal tibia. Identify the medial collateral ligament (MCL).

Place a 3.0 mm K-wire (jig pin), as a jig pin through the proximal jig pin hole in the arm of the jig.

Ensure the hole is clear by rotating the jig pin screw counter-clockwise. The screw does not need to be completely removed. The hole is clear once any threads are observed as the jig pin screw is loosened.

Insert the jig pin 3 mm–4 mm distal to the joint surface and just caudal to the medial collateral ligament.

It is important to ensure the jig pin is inserted parallel to the articular surface and frontal plane of the tibia and perpendicular to the sagittal plane.

Do not tighten the proximal jig pin screw until after the distal jig pin is inserted.

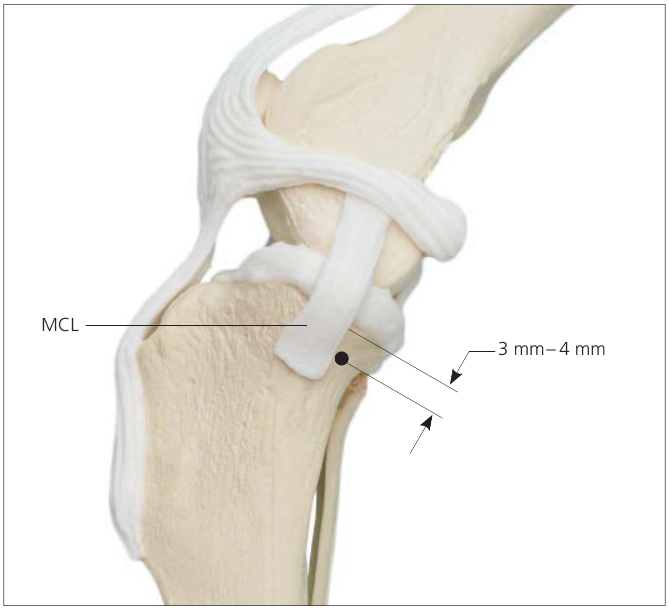

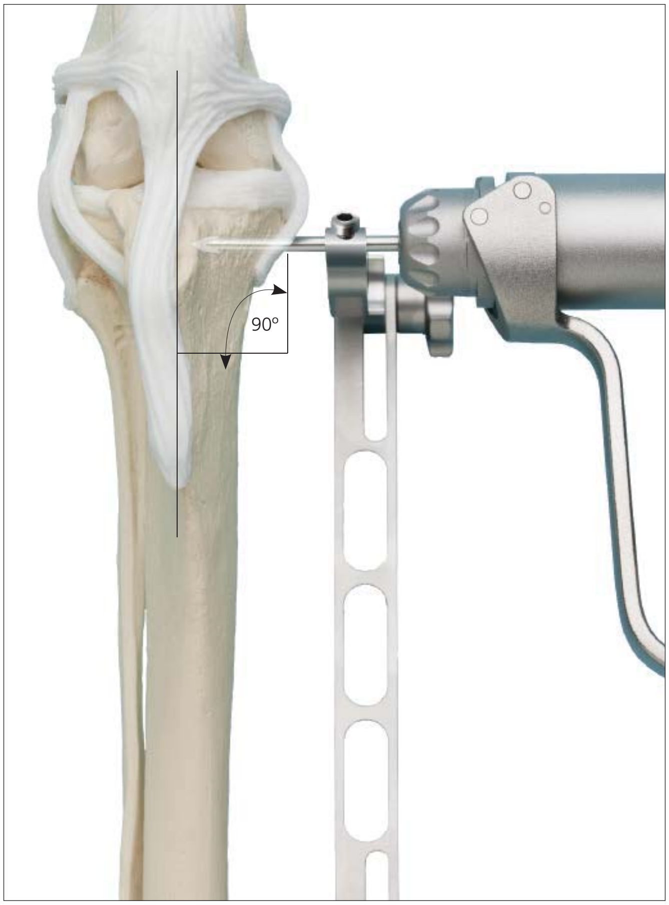

2
Insert Distal Jig Pin

Instruments	
VW3001.15	3.0 mm Kirschner Wire, 150 mm (jig pin)
314.02	Small Hexagonal Screwdriver with Holding Sleeve

Place a 3.0 mm K-wire jig pin through the distal jig pin hole in the arm of the jig.

Insert the pin ensuring it is parallel to the proximal jig pin and centered in the tibia.

Tighten the jig pin screws with the small hexagonal screwdriver.

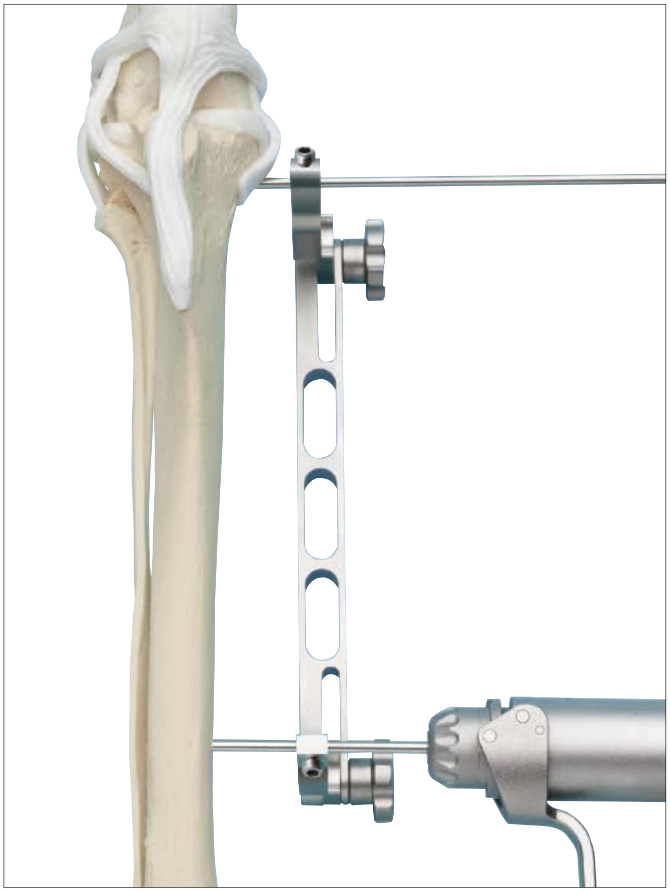

3
Cut Proximal Jig Pin

Instrument	
388.72	Rod Cutter

To provide clearance for the saw blade, cut the proximal jig pin leaving no more than 6 mm–8 mm protruding above the jig.

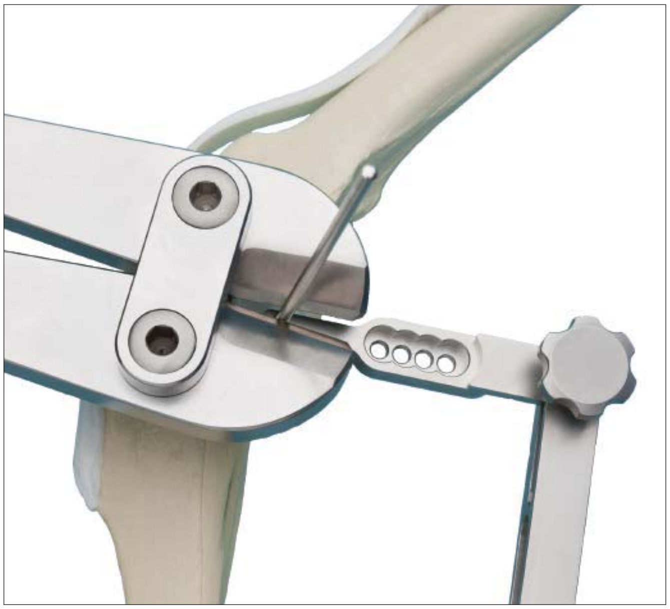

4
Attach Saw Guide

Instruments

VQ0001.24	24 mm Saw Guide
or	
VQ0001.27	27 mm Saw Guide
or	
VQ0001.30	30 mm Saw Guide

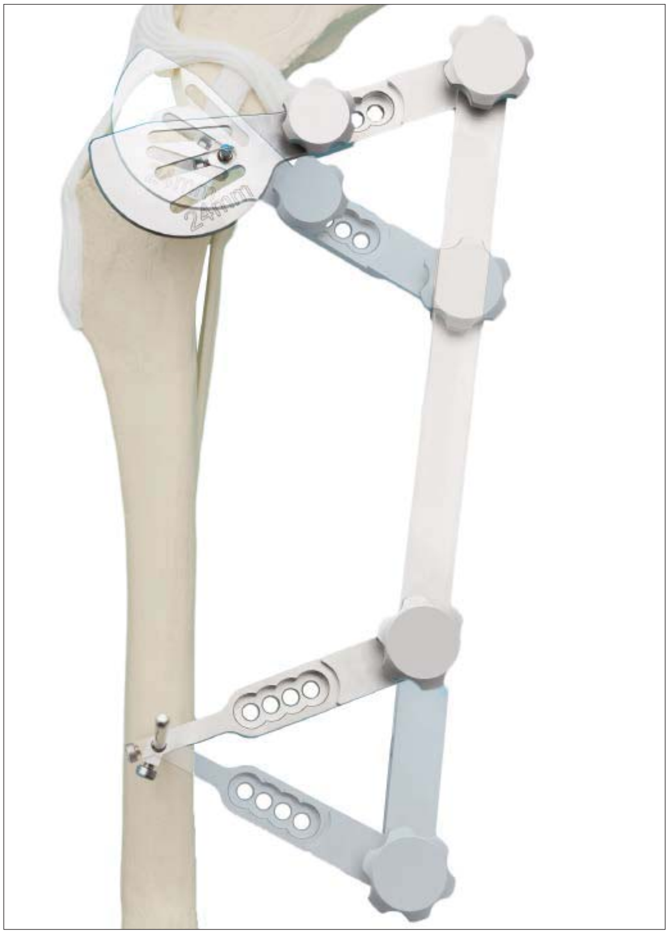

Attach the appropriate saw guide based on preoperative planning. Select the optimal position for the saw guide. There are 4 screw positions and 3 angular positions for the saw guide (12 total).

Additional adjustment of the saw guide position can be made by angulating the jig arms, as shown.

Confirm optimal position of the planned osteotomy by placing the saw in proper alignment against the saw guide. Ensure that the axis of the saw is parallel to the jig pins.

Note: **In medium-to-large breed dogs, it is recommended that the osteotomy leave a tibial tuberosity width of at least 10 mm or greater to avoid tuberosity fracture postoperatively.**

Once the proper osteotomy position is determined, securely tighten the jig pin and hinge screws.

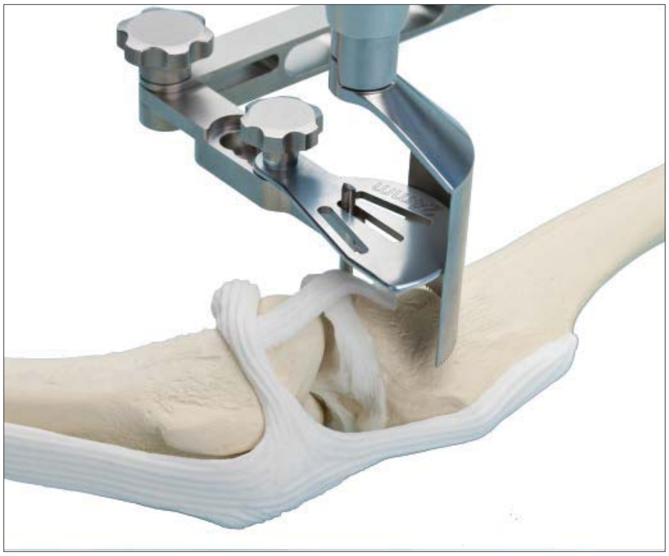

5

Perform Osteotomy

Instruments

03.000.394	Crescentic Saw Blade, 24 mm radius, 45 mm x 0.6 mm
03.000.395	Crescentic Saw Blade, 27 mm radius, 50 mm x 0.6 mm
03.000.396	Crescentic Saw Blade, 30 mm radius, 50 mm x 0.6 mm
399.82	Osteotome, 10 mm/150 mm
532.110	Small Battery Drive II
532.026	Oscillating Saw Attachment, large

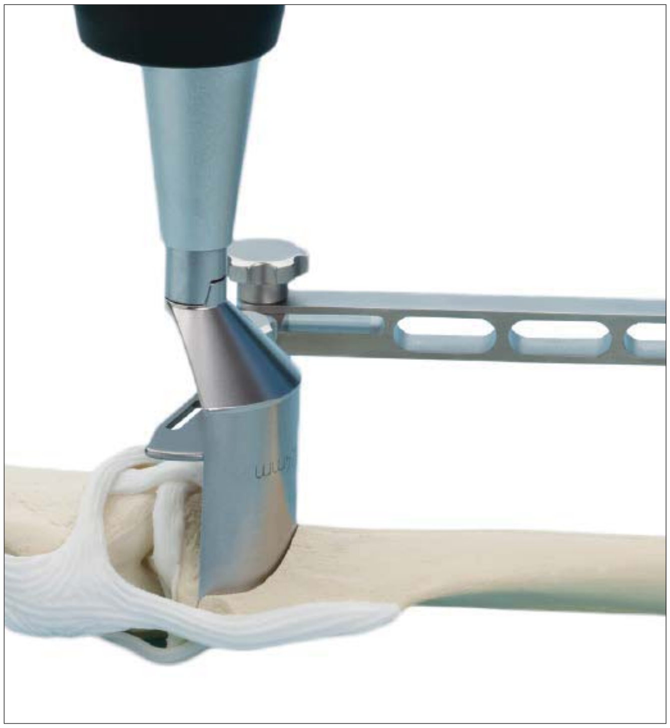

Perform a partial osteotomy of the proximal tibia. The cut is made approximately half-way through the bone. Care must be taken to ensure the cut is made parallel to the distal jig pin.

Remove the saw guide.

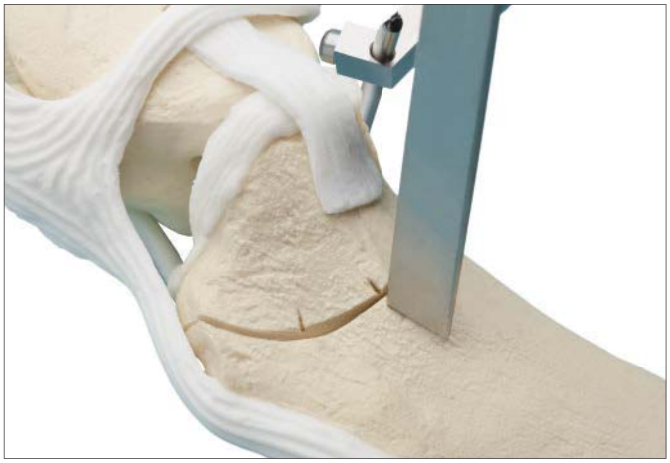

Place a mark on the proximal bone fragment near the edge of the osteotomy. This mark should be located cranial to the midpoint of the osteotomy.

Make a second mark on the proximal bone fragment at the proper distance from the first mark. This distance should be determined from the TPLO Rotation Quick Reference Chart (page 34).

Transfer the second mark across the cut, to the distal bone fragment.

Complete the osteotomy.

Note: **The saw guide is no longer necessary, since the osteotomy location and saw orientation have already been determined by the initial cut.**

6

Rotate Proximal Bone Fragment

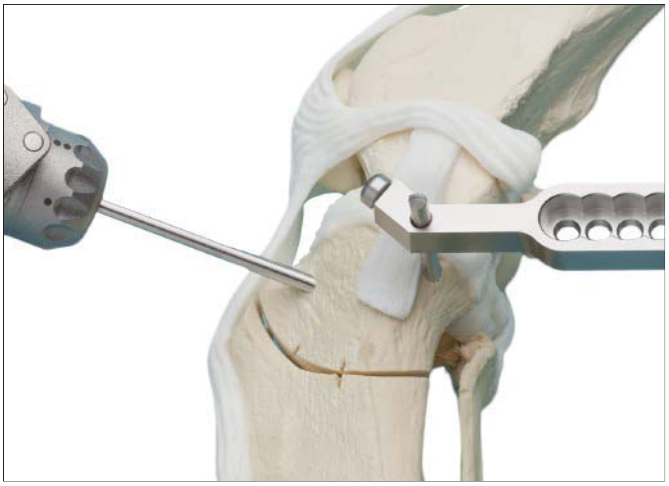

Instruments

VW1203.15	1.25 mm Kirschner Wire, 150 mm
VW3001.15	3.0 mm Kirschner Wire, 150 mm (pin)

Insert a 3.0 mm K-wire (pin), or larger, into the proximal bone fragment at an oblique angle, above the level of the patellar tendon insertion. Orient the pin to avoid the articular surface and osteotomy and aim just below the jig pin, while ensuring penetration into the far cortex.

Rotate the proximal bone fragment to align the marks.

Note: Do not attempt to align the medial surfaces of the bone. A small step can be expected.

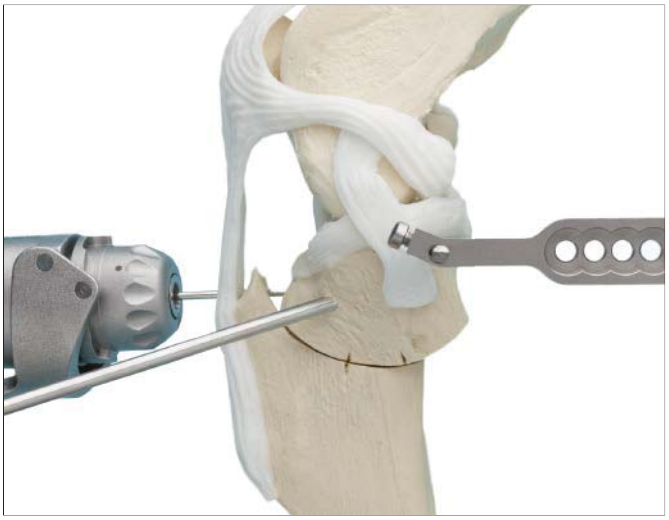

Secure the tibial plateau segment in the rotated position by inserting a 1.25 mm K-wire beginning proximolateral to the patellar tendon insertion on the tibial tuberosity and through the tibial plateau segment. This K-wire should be aimed just distal to the jig pin.

Remove the K-wire (pin) used for rotation.

Tip: Application of large pointed reduction forceps from the tibial tuberosity to the caudal margin of the tibial plateau provides additional stability of the osteotomy.

7

Place Plate and Contour

Place plate on bone and contour as required (if using conventional screws). Observe precautions described on page 11 when contouring the plate.

SCREW INSERTION SEQUENCE

The following technique is shown using the 3.5 mm TPLO plate. The same procedure should be used for 3.5 mm small and 2.7 mm. It is recommended that screws be inserted in the sequence described below:

1

In the proximal DCP shaft hole, place a conventional cortex screw in neutral position.

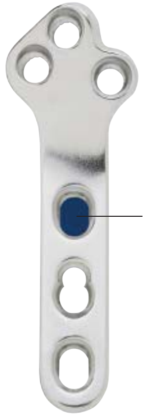

2

In the most distal DCP shaft hole, place a conventional cortex screw in the load position. This screw should be left slightly loose, by one turn (i.e., the screw head is not placed into the plate hole.)

Note: **When rotational correction is performed, this screw should be placed in neutral position.**

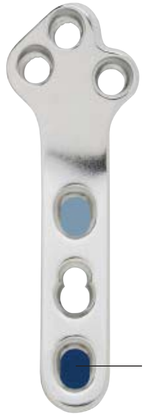

3

Place either a conventional cortex screw or locking screw in the most cranial head hole of the plate. Fully tighten this screw. If both cortex and locking screws are used in the plate head, place and tighten all cortex screws first and then place all locking screws.

Note: **Do not lock the screws to the plate under power. Screw head thread engagement and final locking torque must be performed manually. The following Torque Limiting Attachments are indicated for their respective TPLO Systems:**

- **For 3.5 mm Locking Screws, 511.773 Torque Limiting Attachment, 1.5 Nm, quick coupling**
- **For 2.7 mm Locking Screws, 511.776 Torque Limiting Attachment, 0.8 Nm, quick coupling**

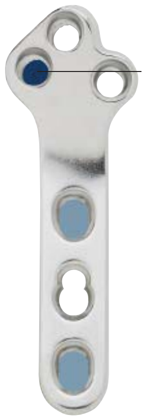

*See page 28 for the 0.8 Nm and 1.5 Nm Torque Limiting Attachment.

4

Place a second conventional screw or locking screw in the head of the plate. Select the most easily accessible plate hole, avoiding the jig and holding K-wire. If necessary, articulate the jig arms to gain access. The jig should remain in place until at least 2 screws have been inserted into the plate head. Fully tighten this screw.

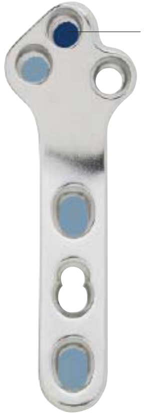

5

Tighten the most distal shaft screw, until it makes initial contact with the plate/DCP hole.

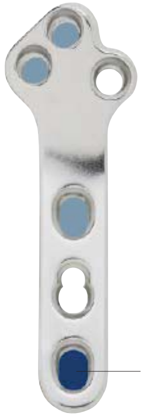

6

Slightly loosen the screw in the proximal DCP shaft hole. Fully tighten the screw in the most distal DCP shaft hole.

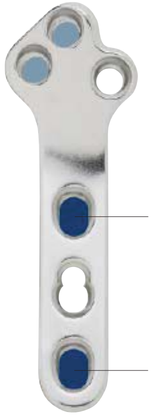

7

Retighten the screw in the proximal DCP shaft hole.

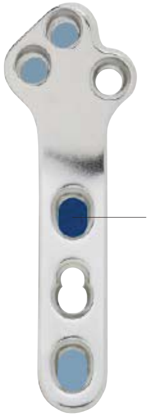

8

Remove jig. Place either a conventional screw or locking screw in the last head hole of the plate. Fully tighten this screw.

Note: It is highly recommended that at least 2 locking screws be used in the proximal, head portion of the TPLO plate (Steps 3, 4, and 8).

In the fourth head hole in the 3.5 mm broad plate, place either a conventional screw or locking screw.

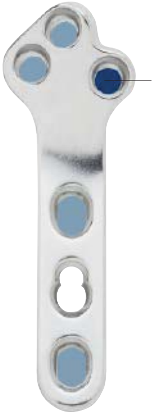

9

Place either a conventional cortex screw in the neutral position in the non-threaded portion of the LCP Combi hole or a locking screw in the threaded portion of the LCP Combi hole. Fully tighten this screw.

Check tightness of all screws placed previously.

In the fourth shaft hole in the 3.5 mm broad plate, place either a conventional cortex screw in the neutral position in the non-threaded portion of the Combi hole or a locking screw in the threaded portion of the Combi hole.

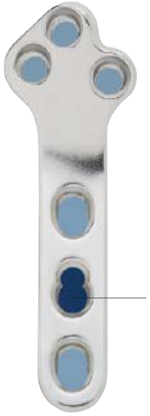

IMPLANTS

2.7 mm Tibial Plateau Leveling Osteotomy (TPLO) Plate,
46 mm long, 2.5 mm thick

	Proximal Holes	Distal Holes	
VP4400.L3	3	3	left
VP4400.R3	3	3	right

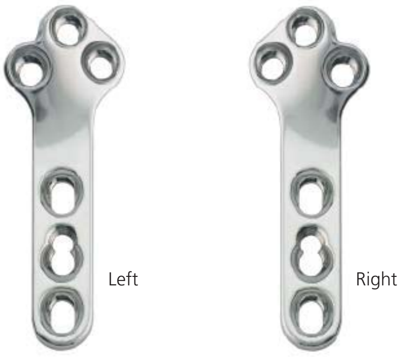

3.5 mm Small Tibial Plateau Leveling Osteotomy (TPLO) Plate,
55 mm long, 3.7 mm thick

	Proximal Holes	Distal Holes	
VP4403.L3	3	3	left
VP4403.R3	3	3	right

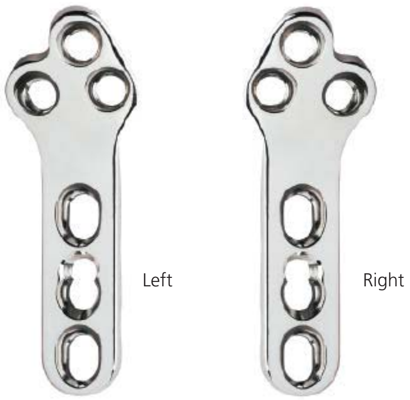

3.5 mm Tibial Plateau Leveling Osteotomy (TPLO) Plate,
64 mm long, 3.7 mm thick

	Proximal Holes	Distal Holes	
VP4401.L3	3	3	left
VP4401.R3	3	3	right

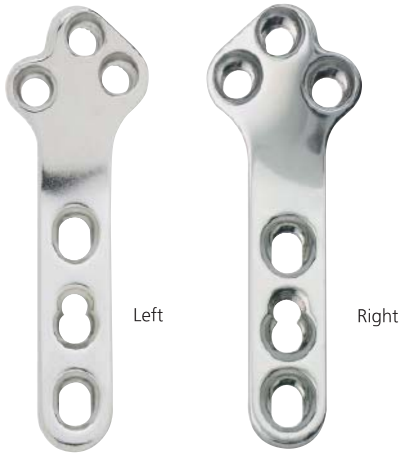

3.5 mm Broad Tibial Plateau Leveling Osteotomy (TPLO) Plate,
80 mm long, 3.7 mm thick

	Proximal Holes	Distal Holes	
VP4402.L4	4	4	left
VP4402.R4	4	4	right

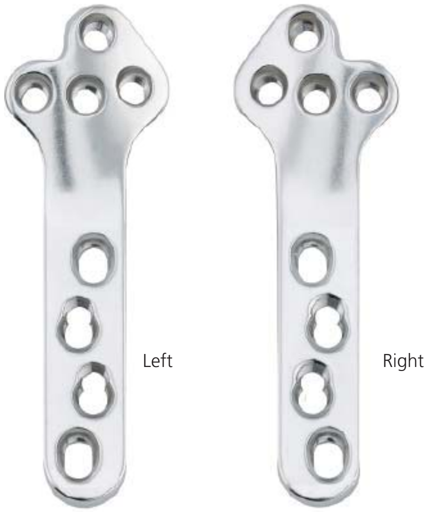

2.7 mm Cortex Screws, self-tapping

VS205.006–VS205.044	6 mm–44 mm (in 2 mm increments)
VS205.045–VS205.055	45 mm–55 mm (in 5 mm increments)

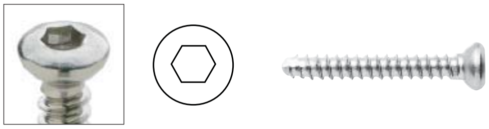

2.7 mm Locking Screws, self-tapping, with StarDrive recess

VS206.010–VS206.034	10 mm–34 mm (in 2 mm increments)

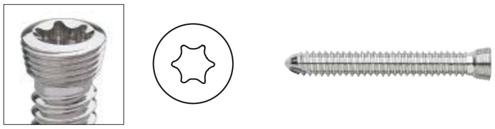

3.5 mm Locking Screws, self-tapping, with StarDrive recess

VS301.010–VS301.040	10 mm–40 mm (in 2 mm increments)
VS301.045–VS301.070	45 mm–70 mm (in 5 mm increments)

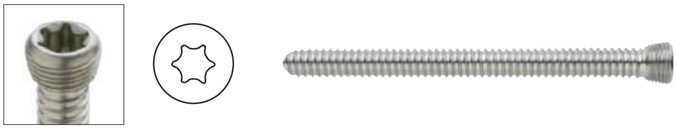

3.5 mm Cortex Screws, self-tapping

VS302.010–VS302.050	10 mm–50 mm (in 2 mm increments)
VS302.055–VS302.070	55 mm–70 mm (in 5 mm increments)

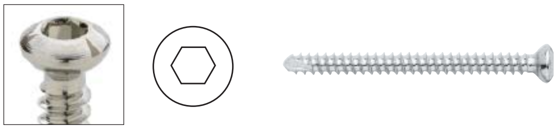

Screw Reference Chart

Thread Diameter	**2.7 mm**	**2.7 mm**	**3.5 mm**	**3.5 mm**	**4.0 mm**
Screw Type	**Cortex**	**Locking**	**Cortex**	**Locking**	**Cancellous**
Drill Bit for Threaded Hole	2.0 mm	2.0 mm	2.5 mm	2.8 mm	2.5 mm
Tap	2.7 mm	Self-Tapping	3.5 mm	Self-Tapping	4.0 mm
Drive Type	2.5 mm Hexagonal	T8 StarDrive	2.5 mm Hexagonal	T15 StarDrive	2.5 mm Hexagonal

2.7 mm Cortex Screws, non-self tapping

VS204.006–VS204.040	6 mm–40 mm (in 2 mm increments)
VS204.045–VS204.055	45 mm–55 mm (in 5 mm increments)

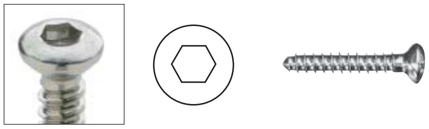

3.5 mm Cortex Screws, non-self tapping

VS301.010–VS301.040	10 mm–40 mm (in 2 mm increments)
VS301.045–VS301.070	45 mm–70 mm (in 5 mm increments)

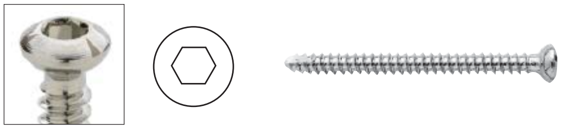

4.0 mm Cancellous Bone Screws, fully threaded

VS403.010–VS403.032	10 mm–32 mm (in 2 mm increments)
VS403.035–VS403.070	35 mm–70 mm (in 5 mm increments)

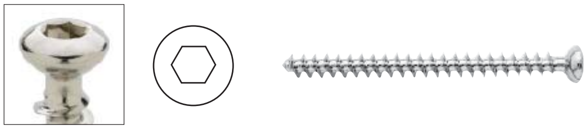

4.0 mm Cancellous Bone Screws, partially threaded

VS404.010–VS404.030	10 mm–30 mm (in 2 mm increments)
VS404.035–VS404.070	35 mm–70 mm (in 5 mm increments)

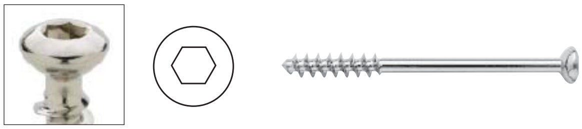

Also Available

VP4405.L3	2.0 mm Tibial Plateau Leveling Osteotomy (TPLO) Plate, left
VP4405.R3	2.0 mm Tibial Plateau Leveling Osteotomy (TPLO) Plate, right
VP4404.L3	2.4 mm Tibial Plateau Leveling Osteotomy (TPLO) Plate, left
VP4404.R3	2.4 mm Tibial Plateau Leveling Osteotomy (TPLO) Plate, right

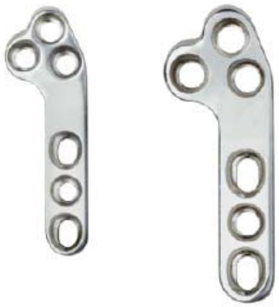

INSTRUMENTS

310.21	2.0 mm Drill Bit, quick coupling, 125 mm	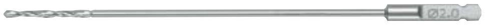
310.288	2.8 mm Drill Bit, quick coupling, 165 mm	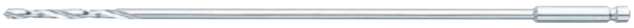
312.648	2.8 mm Threaded Drill Guide	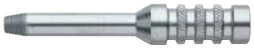
313.353	2.0 mm LCP Solid Threaded Drill Guide	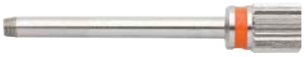
314.115	StarDrive Screwdriver, T15	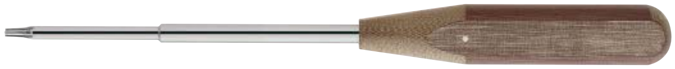
314.116	StarDrive Screwdriver Shaft, T15, quick coupling	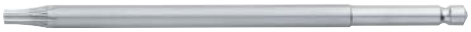

314.467	StarDrive Screwdriver Shaft, T8	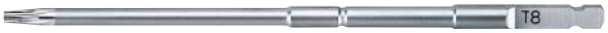
323.26	2.7 mm Universal Drill Guide	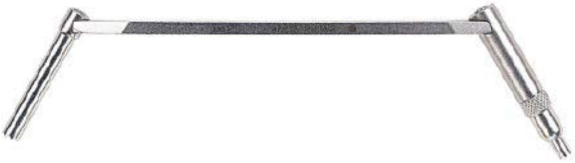
323.36	3.5 mm Universal Drill Guide	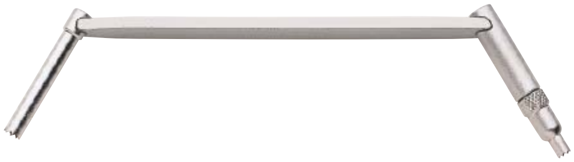
324.023	Threaded Plate Holder	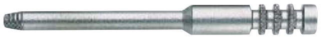

Also Available

511.773	Torque Limiting Attachment, quick coupling, 1.5 Nm	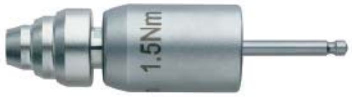
511.776	Torque Limiting Attachment, quick coupling, 0.8 Nm	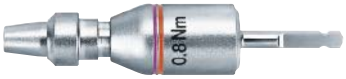

VQ0001.00	Standard TPLO Jig	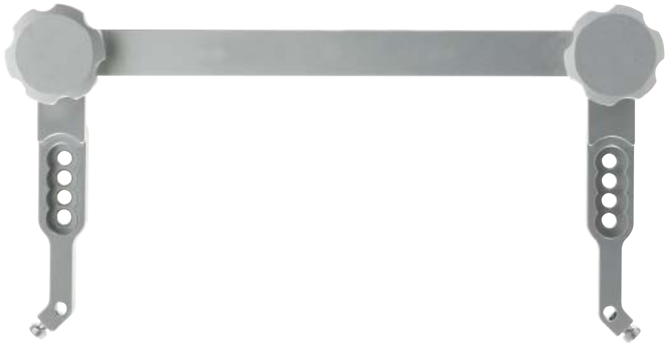

	Saw Guides, for use with Standard TPLO Jig	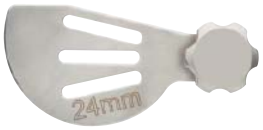
VQ0001.24	24 mm	
VQ0001.27	27 mm	
VQ0001.30	30 mm	

Also Available

VQ0001.02	Replacement Hinge Screw	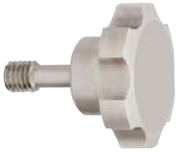
VQ0001.03	Replacement Saw Guide Screw	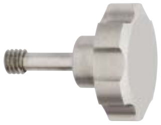
VQ0001.04	Long Replacement Jig Pin Screw	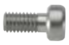

Also Available

VW1203.15	1.25 mm Kirschner Wire, 150 mm

VW3001.15	3.0 mm Kirschner Wire with Trocar Point, 150 mm

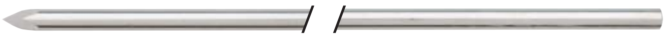

388.72	Rod Cutter

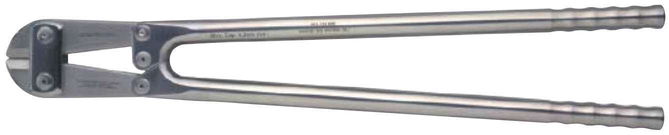

	Osteotomes, 150 mm length
399.80	2 mm width
399.81	5 mm width
399.82	10 mm width

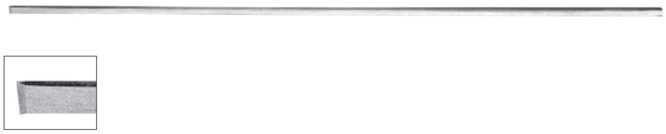

Small Battery Drive II and Accessories

05.001.204	Universal Battery Charger II
05.001.250	AO/ASIF Quick Coupling for Drill Bits
532.104	Battery Insertion Shield
532.110	Small Battery Drive II
532.022	Quick Coupling for K-wires
532.026	Oscillating Saw Attachment, large
532.027	Replacement Key, for 532.026
532.132	Battery Casing for 14.4 V Li Ion Battery
532.103	Small Battery Drive 14.4 V Li Ion Battery

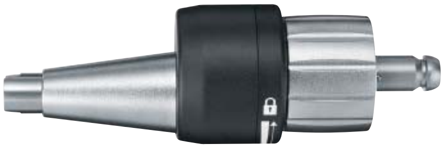

Crescentic Saw Blades

03.000.391	15 mm radius, 45 mm x 0.6 mm
03.000.392	18 mm radius, 45 mm x 0.6 mm
03.000.393	21 mm radius, 45 mm x 0.6 mm
03.000.394	24 mm radius, 45 mm x 0.6 mm
03.000.395	27 mm radius, 50 mm x 0.6 mm
03.000.396	30 mm radius, 50 mm x 0.6 mm

Note: **All sawblades come with attachment screws.**

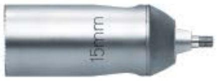

03.000.391

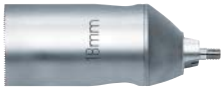

03.000.392

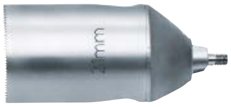
03.000.393

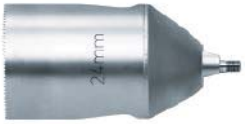

03.000.394

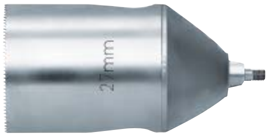

03.000.395

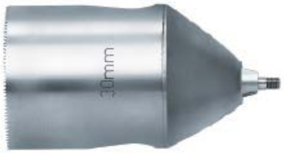

03.000.396

For the full range of attachments and accessories for the Small Battery Drive II, please contact your DePuy Synthes representative or consult the DePuy Synthes Power Tools product catalog.

SET INFORMATION

Recommended Sets

103.503	Small Fragment Instrument Set, Veterinary
103.515	Small Fragment Screw Set, Veterinary

Note: Small Fragment Instrument Set (103.503) consists of Standard Instrument Set (103.501), graphic case, and Locking Instrument Set (103.502).

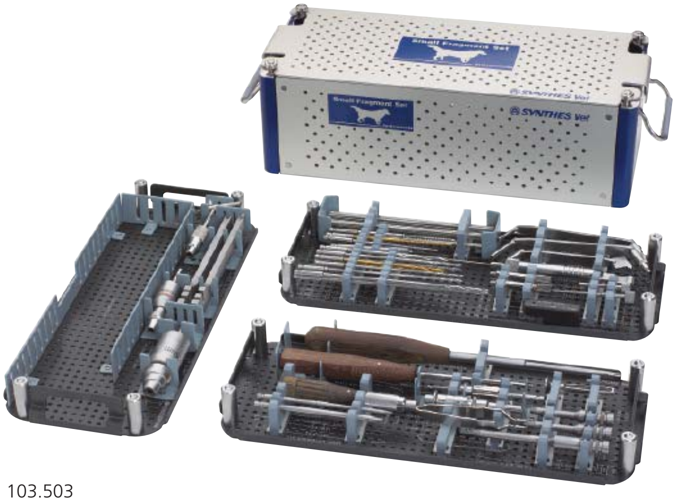

103.503

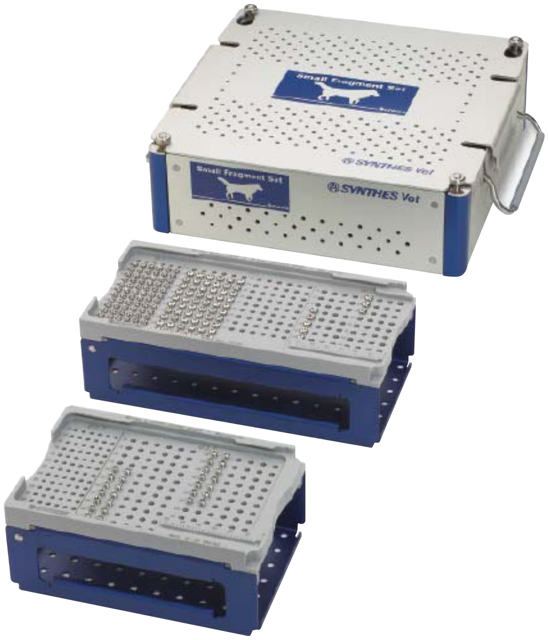

103.515

For detailed cleaning and sterilization instructions, please refer to:
www.synthes.com/cleaning-sterilization
In Canada, the cleaning and sterilization instructions will be provided with the Loaner shipments.

Optional Storage Options

690.590 Tibial Plateau Leveling Osteotomy (TPLO) Plate Set Graphic Case

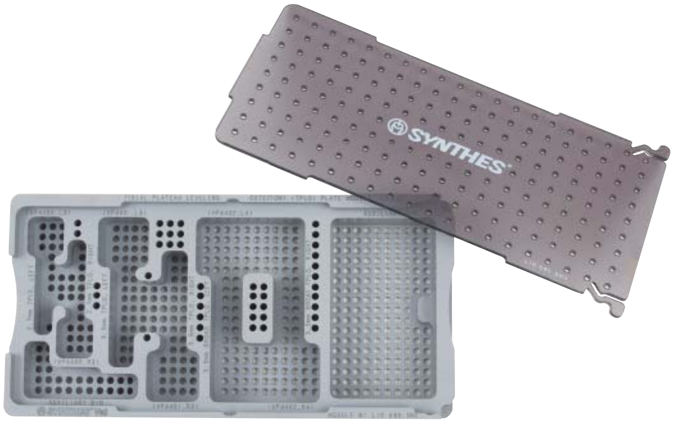

690.590

TPLO ROTATION

Quick Reference Chart

PREOPERATIVE TIBIAL PLATEAU ANGLE (TPA)

Saw Radius	15º	16º	17º	18º	19º	20º	21º	22º	23º	24º	25º	26º	27º
**12 mm**	2.0	2.2	2.4	2.6	2.9	3.1	3.3	3.5	3.7	3.9	4.1	4.3	4.5
**15 mm**	2.6	2.8	3.1	3.3	3.6	3.8	4.1	4.3	4.6	4.9	5.1	5.4	5.6
**18 mm**	3.1	3.4	3.7	4.0	4.3	4.6	4.9	5.2	5.5	5.8	6.1	6.5	6.8
**21 mm**	3.6	4.0	4.3	4.7	5.0	5.4	5.8	6.1	6.5	6.8	7.2	7.5	7.9
**24 mm**	4.1	4.5	5.0	5.4	5.8	6.2	6.6	7.0	7.4	7.8	8.2	8.6	9.0
**27 mm**	4.7	5.1	5.6	6.0	6.5	7.0	7.4	7.9	8.4	8.8	9.3	9.7	10.2
**30 mm**	5.2	5.7	6.2	6.7	7.2	7.8	8.3	8.8	9.3	9.8	10.3	10.8	11.3

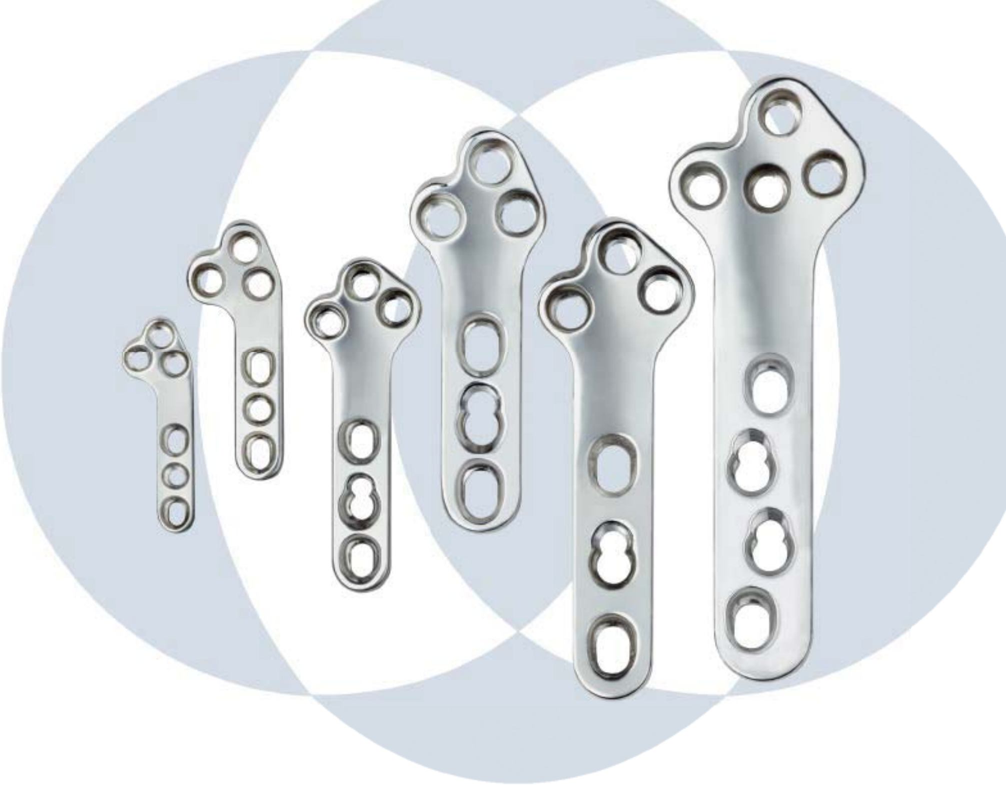

28°	29°	30°	31°	32°	33°	34°	35°	36°	37°	38°	39°	40°
4.7	4.9	5.1	5.3	5.5	5.7	5.9	6.1	6.3	6.4	6.6	6.8	7.0
5.9	6.1	6.4	6.6	6.9	7.1	7.4	7.6	7.9	8.1	8.4	8.6	8.8
7.1	7.4	7.7	8.0	8.3	8.6	8.9	9.2	9.5	9.8	10.1	10.3	10.6
8.3	8.6	9.0	9.3	9.7	10.0	10.4	10.7	11.1	11.4	11.8	12.1	12.4
9.5	9.9	10.3	10.7	11.1	11.5	11.9	12.3	12.7	13.1	13.5	13.9	14.3
10.6	11.1	11.6	12.0	12.5	12.9	13.4	13.8	14.3	14.7	15.2	15.6	16.1
11.8	12.3	12.9	13.4	13.9	14.4	14.9	15.4	15.9	16.4	16.9	17.4	17.9

Limited Warranty and Disclaimer: DePuy Synthes Vet products are sold with a limited warranty to the original purchaser against defects in workmanship and materials. Any other express or implied warranties, including warranties of merchantability or fitness, are hereby disclaimed.

WARNING: In the USA, this product has labeling limitations. See package insert for complete information.

CAUTION: USA Law restricts these devices to sale by or on the order of a physician.

Not all products are currently available in all markets.

DePuy Synthes VET

COMPANIES OF Johnson & Johnson

DePuy Synthes Vet
1302 Wrights Lane East
West Chester, PA 19380
Telephone: (610) 719-5000
To order: (800) 523-0322

www.synthesvet.com

© DePuy Synthes Vet, a division of DOI 2013. All rights reserved.

J6544-C 1/14
