# Supplementary material for: Novel TPLO Alignment Jig/Saw Guide Reproduces Freehand and Ideal Osteotomy Positions
Source: PLoS One. 2016 Aug 24;11(8):e0161110. doi: 10.1371/journal.pone.0161110 (PMC4996453; doi:10.1371/journal.pone.0161110)
Supplement: S3 File — (PDF) [file pone.0161110.s003.pdf]

Tufts UNIVERSITY | Cummings School of Veterinary Medicine

Dear Ms. Cunningham:

We previously requested approval from DPS to publish the figure below (and have attached your [DPS] previous approval), but are being asked to provide specific approval according to the Creative Commons Attribution License (CCAL) CC BY 4.0 (http://creativecommons.org/licenses/by/4.0/). I have included on the follow page the wording that was requested – both in terms of the request and the changes for the figure legend. The manuscript has been submitted to PlosOne, entitled: "Novel TPLO Alignment Jig/Saw Guide Reproduces Freehand and Ideal Osteotomy Positions", authored by Mariano AD, Kowaleski MP and Boudrieau RJ. I would appreciate your sending me your approval again such that it conforms to the requested requirements.

Thank you.

Sincerely yours,

Randy J Boudrieau

Digitally signed by Randy J Boudrieau
DN: cn=Randy J Boudrieau, o=Cummings School of Veterinary Medicine at Tufts University, ou=Dept. of Clinical Sciences, email=randy.boudrieau@tufts.edu, c=US
Date: 2016.07.29 08:59:34 -04'00'

Dr. Randy J. Boudrieau, Diplomate ACVS and ECVS
Professor of Surgery
Cummings School of Veterinary Medicine
Tufts University
Department of Clinical Sciences
200 Westboro Road
North Grafton, MA 01536

Hospital	508-839-5395
Dept. Office	508-839-7960
Dept. Fax	508-839-7922

I request permission for the open-access journal PLOS ONE to publish the following figure below under the Creative Commons Attribution License (CCAL) CC BY 4.0 (http://creativecommons.org/licenses/by/4.0/). Please be aware that this license allows unrestricted use and distribution, even commercially, by third parties. Please reply and provide explicit written permission to publish this figure under a CC BY license.

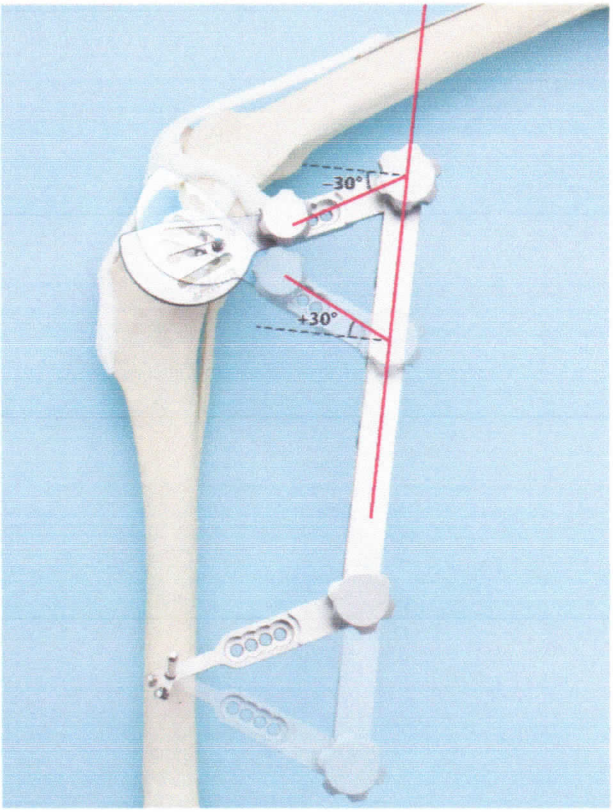

Figure legend:

After the saw guide was placed in the closest position to the osteotomy, the jig arms were angulated to achieve the best guide-to-osteotomy fit. The baseline angle of the jig arm was 90°. Angles greater than 90° were recorded as positive and angles below 90° were recorded as negative. The magnitude of the angle was recorded as its total deviation from 90°. Reprinted from the DePuy Synthes Vet Technique Guide: "Standard Tibial Plateau leveling Osteotomy (TPLO) System" (J6544-C, 2013) under a CC BY license, with the permission of DePuy Synthes Vet, Inc. West Chester, PA; USA
