# Supplementary material for: Novel TPLO Alignment Jig/Saw Guide Reproduces Freehand and Ideal Osteotomy Positions
Source: PLoS One. 2016 Aug 24;11(8):e0161110. doi: 10.1371/journal.pone.0161110 (PMC4996453; doi:10.1371/journal.pone.0161110)
Supplement: S2 File — (PDF) [file pone.0161110.s002.pdf]

DePuy Synthes VET

COMPANIES OF Johnson&Johnson

April 21, 2016

Dr. Randy J. Boudrieau
Professor of Surgery
Dept. of Clinical Sciences
Tufts Cummings School of Veterinary Medicine
200 Westboro Rd.
North Grafton, MA 01536

Re: Permission Request

Dear Dr. Boudrieau,

We are in receipt of your request for permission to use the embedded image (the "Image") in an article, currently entitled **Novel TPLO alignment jig/saw guide reproduces freehand and ideal osteotomy position**, which will be **submitted for publication in PLOS ONE** (the "Publication").

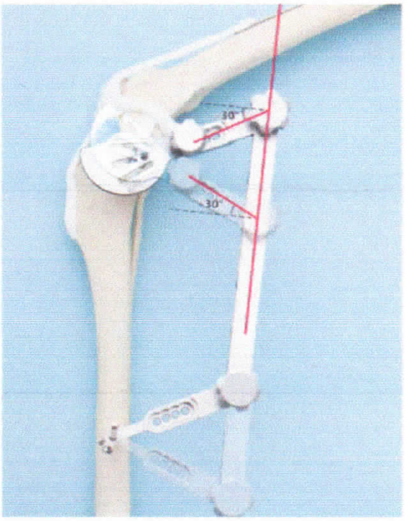

DePuy Synthes Vet hereby grants Dr. Randy J. Boudrieau non-exclusive permission to use the Image in the Publication under the following conditions:

DePuy Synthes VET

COMPANIES OF Johnson&Johnson

1. The copyrights in the Images remain owned by DePuy Synthes.
2. The following image caption must be included: "Courtesy of DePuy Synthes Vet".
3. The use of the Image may not promote off-label use.
4. The Image may not be changed or edited in any way, unless approved in writing by DePuy Synthes Vet.
5. This permission may not be transferred, sublicensed or assigned to any third party.
6. DePuy Synthes Vet shall have the right to withdraw this permission at any time by providing written notice to Dr. Randy J. Boudrieau.

DePuy Synthes Vet represents and warrants that it has the right to grant the permissions contained herein.

Again, we appreciate your interest in DePuy Synthes Vet.

I acknowledge that I have read this agreement and accept the terms and conditions stated.

Name/Signature Randy J Boudrieau

Date

Digitally signed by Randy J Boudrieau
DN: cn=Randy J Boudrieau, o=Cummings School of Veterinary Medicine at Tufts University, ou=Dept. of Clinical Sciences, email=randy.boudrieau@tufts.edu, c=US
Date: 2016.04.21 13:32:28 -04'00'

Very truly yours,

Horan, Timothy [SYNNA]

Digitally signed by Horan, Timothy [SYNNA]
DN: cn=Horan, Timothy [SYNNA], o, ou, email=thoran@its.jnj.com, c=US
Date: 2016.04.22 08:02:12 -04'00'

Timothy J. Horan
Staff Engineer, DePuy Synthes Vet
